# Leader–Member Exchange Fosters Beneficial and Prevents Detrimental Workplace Behavior: Organizational Identification as the Linking Pin

**DOI:** 10.3389/fpsyg.2020.01788

**Published:** 2020-08-18

**Authors:** Martin Götz, Michelle Donzallaz, Klaus Jonas

**Affiliations:** ^1^Department of Psychology, University of Zurich, Zurich, Switzerland; ^2^Department of Psychology, University of Amsterdam, Amsterdam, Netherlands

**Keywords:** leader–member exchange, organizational identification, norms, workplace deviance, multi-methods research, organizational citizenship behavior (OCB), counterproductive work behavior (CWB)

## Abstract

Discretionary behaviors, such as counterproductive work behavior (CWB) and organizational citizenship behavior (OCB), directly refer to an organization’s normative expectations. As such, employees engaging in these behaviors violate or exceed organizational norms, respectively. An employee’s relationship quality with his or her supervisor [i.e., leader–member exchange (LMX)] has been found to be a prominent antecedent of employees’ workplace behavior. However, the actual mechanisms that link LMX and discretionary behaviors (i.e., CWB and OCB) are not yet well understood. Integrating social exchange as well as the social identity theory, we present an employee’s organizational identification (OI) as a mechanism that sheds light on why LMX leads to employees’ subsequent discretionary behavior. Across four empirical studies employing complementary study designs, we demonstrate that LMX is positively associated with OI, which, in turn, curbs CWB and fosters OCB. Specifically, this pattern of findings is consistent across (1) a cross-sectional study with 188 Swiss employees, (2) a time-lagged study with 502 Swiss employees, (3) an online recall experiment with 131 US participants, and (4) an online vignette experiment with 139 US participants. In sum, we present an integrative theoretical model and respective empirical support to shed light on OI as a pivotal mechanism that can explain why the relationship quality with one’s supervisor can simultaneously serve as a deterrent for CWB and foster OCB.

## Introduction

*“Tend to the people, and they will tend to the business.”* John C.

Maxwell (2011)

In 2014, the largest retail pharmacy in the United States, CVS, faced 29 million USD in fines for losing track of painkillers, suggesting that CVS employees stole prescription drugs (Lazarus, 2014). Such example illustrates how critical it is to understand why some employees harm their employer by violating organizational norms [i.e., counterproductive work behavior (CWB); O’Boyle et al., 2011; Mercado et al., 2018; Götz et al., 2019]. At the same time, it is of equal importance to understand why some employees exceed organizational norms in a positive fashion by going the *extra mile* [i.e., organizational citizenship behavior (OCB); e.g., Organ et al., 2006; Podsakoff et al., 2018; Spitzmüller et al., 2018].

An important factor that determines how employees feel and behave at the workplace is the relationship quality with their immediate supervisors [i.e., leader–member exchange (LMX); e.g., Scandura and Graen, 1984; Liden et al., 1997; Bauer and Erdogan, 2015]. Research consistently demonstrated the pivotal role of LMX with regard to subordinates’ reactions and behaviors [for reviews, see Schriesheim et al. (1999), Martin et al. (2010), and Anand et al. (2011)]. Specifically regarding employees’ CWB and OCB, meta-analytic evidence illustrates that when employees perceive the relationship with their supervisor to be of high quality, they are less likely to engage in CWB, while they are also more inclined to display OCB (e.g., Gerstner and Day, 1997; Dulebohn et al., 2012; Martin et al., 2016).

While the relationships between LMX and CWB as well as OCB are rather well established, little is known about the underlying mechanisms, particularly regarding the relationship of LMX and CWB (Martin et al., 2010, 2016). We draw from the social identity approach (e.g., Tajfel and Turner, 1986; Turner et al., 1987; Haslam, 2004) to argue that the extent to which employees identify with their organization [organizational identification (OI)] accounts for the effects of LMX on discretionary workplace behavior, such as CWB and OCB. Employees tend to generalize the relationship quality with their supervisor to the organization (e.g., Gerstner and Day, 1997; Zhang and Chen, 2013; Eisenberger et al., 2019), that is, the better the relationship with their supervisors, the more connected employees feel with their employer. Consequently, they should be more inclined to define themselves in terms of the organization (e.g., Carmeli et al., 2011; Loi et al., 2014; Zhao et al., 2019) and, in turn, act in the organization’s best interest by refraining from CWB and engaging in OCB (e.g., Riketta, 2005; Riketta and Van Dick, 2005; Lee et al., 2015).

**FIGURE 1:**
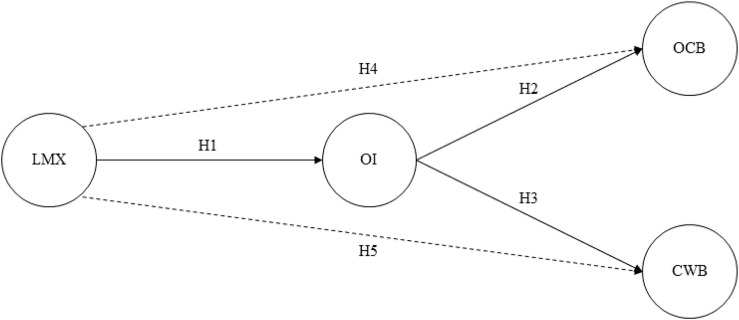
Theoretical model of the current research. Solid lines represent direct effects, whereas dotted lines represent the respective indirect effects. LMX, leader–member exchange; OI, organizational identification; OCB, organizational citizenship behavior; CWB, counterproductive work behavior.

Against this background, we advance theory and research in three ways. First, we present OI as a mechanism underlying the effects of LMX on CWB and OCB, respectively—in doing so, we answer specific calls by Martin et al. (2010, 2016). Second, we extend the literature on social identity by incorporating OI as a central predictor of both CWB and OCB—thereby, we answer specific calls by Lee et al. (2015) as particularly the link of OI and detrimental work behavior (i.e., CWB) is not yet well established empirically [for notable exceptions, see Norman et al. (2010), Al-Atwi and Bakir (2014), and Evans and Davis (2014)]. Third, we present four complementary study designs to test our theoretical model in a robust and triangulating fashion [for methodological in-depth discussions, see Turner et al. (2017), Aguinis et al. (2019), and Podsakoff and Podsakoff (2019)]—in doing so, we offer a consistent empirical support for our theoretical model among employees from Switzerland and the United States in two field studies (studies 1 and 2) as well as in two online experiments (studies 3 and 4; Figure 1).

## Theoretical Background

### Leader–Member Exchange Fosters Organizational Identification

Social exchange processes at the workplace play a pivotal role in establishing desirable attitudinal and behavioral outcomes of employees in organizations—exemplarily, they have been shown to increase job performance and job satisfaction (e.g., Dulebohn et al., 2012, 2017; Martin et al., 2016). At the most general level, the social exchange theory (SET; e.g., Blau, 1964; Cropanzano and Mitchell, 2005; Cropanzano et al., 2017) understands social life as involving a series of sequential transactions of resources between two or more parties. This exchange of resources is governed by the norm of reciprocity in that one party tends to repay the other party in accordance to the value of the exchange (Gouldner, 1960). As such, an employee may choose to reciprocate perceived treatment by the supervisor with respective positive or negative behavior (e.g., Colquitt et al., 2013; Eisenberger et al., 2019; Greco et al., 2019). Within organizations, people develop differentiated social exchange relationships, most prominently with their direct supervisor (e.g., Graen and Uhl-Bien, 1995; Liden and Maslyn, 1998; Cropanzano et al., 2017).

LMX refers to the quality of the social exchange relationship between an employee and the immediate supervisor (e.g., Graen and Uhl-Bien, 1995; Gerstner and Day, 1997; Martin et al., 2016). Specifically, high-quality relationships (i.e., high LMX) highlight long-term exchanges that are trustful, open-ended, spontaneous, and mutually beneficial. Low-quality relationships (i.e., low LMX), on the other hand, are characterized by a lack of mutual trust, by a focus on contract-based obligations, and by endeavors to maintain balanced exchanges across short-term episodic transactions (e.g., Graen and Uhl-Bien, 1995; Liden et al., 1997; Martin et al., 2016). Compared with related constructs derived from social exchange theory [e.g., team-member exchange (TMX)], LMX shows a relatively greater importance in predicting employees’ attitudes and behaviors at the workplace (Banks et al., 2014). Moreover, it has been argued that “the relationship with one’s supervisors [is] a lens through which the entire work experience is viewed” (Gerstner and Day, 1997, p. 840).

Supervisors are considered to be organizational agents (e.g., Kelman, 1958; Rousseau, 1995; Ostroff, 2019) who enact organizational rules and norms—from an employee’s perspective, supervisors are furthermore understood as proxies for the organization (e.g., Rousseau, 1995; Eisenberger et al., 2010, 2019). Therefore, employees might attribute—and thereby generalize—the status of their relationship with their immediate supervisor to the entire organization (e.g., Gerstner and Day, 1997; Martin et al., 2016; Dulebohn et al., 2017). In line with previous research (e.g., Katrinli et al., 2008; Loi et al., 2014; Zhao et al., 2019), we contend that higher levels of mutual trust and support exchanged between employees and their direct supervisors are associated with the degree to which an employee feels connected with the organization. In particular, LMX provides employees with relevant cues, such as respect from their supervisors, that they generalize to the organization and that forms the extent to which they identify with the organization (e.g., Tyler and Blader, 2003; Blader and Tyler, 2009; Zhao et al., 2019). We therefore hypothesize the following:

*Hypothesis 1:* Leader–member exchange is positively associated with organizational identification.

### Organizational Identification Promotes Desirable and Prevents Detrimental Workplace Behavior

OI reflects the psychological state of defining oneself in terms of one’s organization (e.g., Ashforth and Mael, 1989; Haslam, 2004; Haslam and Ellemers, 2006). At its core, OI has been argued to be a “root construct” (Albert et al., 2000, p. 13) that provides a basis for the development of attitudes toward and behaviors at the workplace—beyond work attitudes such as affective commitment or job satisfaction [for meta-analyses, see Riketta (2005), Riketta and Van Dick (2005), and Lee et al. (2015)]. OI is a form of social identification as conceptualized within the social identity approach (SIA; Haslam, 2004).

The SIA—comprising the social identity (Tajfel and Turner, 1986) and self-categorization theory (Turner et al., 1987)—explicates when, how, and why individuals act in a coordinated manner and thereby lends insight into how organizations can achieve their overarching goals. Specifically, a social identity is defined as “that part of the individuals’ self-concept which derives from their knowledge of their membership of a social group (or groups) together with the value and emotional significance of that membership” (Tajfel, 1981, p. 255). The SIA posits that a social identity is activated by contextual cues which shift individual behavior to intergroup behavior (e.g., Tajfel, 1981; Haslam, 2004; Haslam and Ellemers, 2006). Specifically, a shared social identification, such as OI, enables a collective perception and behavior in that people with a salient social identity more readily think and act in terms of their respective group (e.g., Haslam et al., 1997; Haslam, 2004; Haslam and Ellemers, 2006). Accordingly, OI leads organizational attributes, such as values, goals, and, most notably, norms, to become salient, self-defining, and internalized for employees (e.g., Ashforth and Mael, 1989; Haslam and Ellemers, 2006; Ashforth et al., 2008).

With specific regard to normatively defined behaviors at the workplace, CWB and OCB are discretionary workplace behaviors that are considered to deviate from normative organizational expectations in either a negative or a positive way, respectively (e.g., Viswesvaran and Ones, 2000; Bennett and Stamper, 2001; Rotundo and Sackett, 2002). Specifically, CWB is defined as “voluntary behavior that violates significant organizational norms and in so doing threatens the well-being of an organization, its members, or both” (Robinson and Bennett, 1995, p. 556). As such, CWB subsumes a broad array of individual behaviors that have negative implications for the accomplishment of the organization’s goals. In contrast, OCB refers to “individual behavior that is discretionary, not directly or explicitly recognized by the formal reward system, and that in the aggregate promotes the effective functioning of the organization” (Organ, 1988, p. 4). As such, OCB subsumes behaviors that deviate from organizational norms in a positive way which is why OCB is often referred to as *going the extra mile*. Importantly, despite their somewhat antagonistic conceptualization, CWB and OCB should be understood as two distinct constructs that both cover important facets of the overall job performance an employee can display at work (see also Dalal, 2005; Spector et al., 2010; Dalal and Carpenter, 2018).

Although the SIA suggests that employees internalize organizational norms and adhere to them, OI has been shown to also foster behaviors that exceed organizational norms, such as OCB [for meta-analyses, see Riketta (2005), Riketta and Van Dick (2005), and Lee et al. (2015)]. An explanation for this seemingly contradictory finding is offered by the deviance regulation theory (DRT; Blanton and Christie, 2003). The DRT posits “that people try to maintain positive public and private self-images by choosing desirable ways of deviating from social norms and by avoiding undesirable ways of deviating from social norms” (p. 115)—as such, organizationally identified employees may deviate from organizational norms in a positive fashion to enhance their self-image. In contrast, employees that are only weakly or not identified with their organization have been argued to be associated with less adherence to organizational norms and, ultimately, a greater intent to harm the organization (Vadera and Pratt, 2013)—specifically by engaging in CWB, yet, as Lee et al. (2015) noted in their meta-analysis, the empirical basis for this relationship is currently rather sparse [for notable exceptions, see Norman et al. (2010), Al-Atwi and Bakir (2014), and Evans and Davis (2014)].

In light of the theoretical propositions by the SIA (e.g., Tajfel and Turner, 1986; Turner et al., 1987; Haslam, 2004), the DRT (Blanton and Christie, 2003), and previous research (e.g., Norman et al., 2010; Al-Atwi and Bakir, 2014; Evans and Davis, 2014), we expect employees who strongly identify with their organization to act in the organization’s best interest by even exceeding organizational norms (i.e., OCB). We furthermore contend that employees who identify weakly, if at all, with their organization more readily violate organizational norms, thereby displaying CWB; thus, we hypothesize:

*Hypothesis 2:* Organizational identification is positively associated with organizational citizenship behavior.
*Hypothesis 3:* Organizational identification is negatively associated with counterproductive work behavior.

### Organizational Identification as the Linking Pin

Drawing from SET (Blau, 1964; Cropanzano and Mitchell, 2005; Cropanzano et al., 2017) and the SIA (Tajfel and Turner, 1986; Turner et al., 1987; Haslam, 2004) and based on previous research, we have so far hypothesized (1) that employees generalize the quality of their relationship with their immediate supervisor to the entire organization and thereby align their OI accordingly and (2) that OI not only fosters OCB but also prevents CWB because highly identified employees act in the organization’s best interests. Synthesizing our theoretical argumentation and previous findings, we posit that OI is a central underlying mechanism that may explain why LMX tends to prevent CWB and foster OCB (Figure 1).

Ample research suggests that LMX is a central predictor of discretionary workplace behavior (i.e., CWB and OCB), but the underlying mechanisms are less clear (e.g., Gerstner and Day, 1997; Martin et al., 2010, 2016). We contend that one reason why an employee’s relationship quality with the direct supervisor (i.e., LMX) and CWB as well as OCB are associated is an employee’s OI. Employees may generalize a high-quality social exchange relationship with their supervisors to the organization as a whole (e.g., Gerstner and Day, 1997; Sluss and Ashforth, 2007; Eisenberger et al., 2019), which may lead them to feel a sense of connectedness with the whole organization. OI, as “root construct” (Albert et al., 2000, p. 13) of attitudes and behavior in the workplace, connects employees to the organization as a whole. Because of this sense of oneness, employees may be more inclined to act in line with organizational interests by engaging in OCB. Conversely, employees who have a low-quality social exchange relationship with their supervisor may only weakly identify with their organization and therefore be more inclined to engage in CWB (relatedly, see Blanton and Christie, 2003). Thus, we finally hypothesize:

*Hypothesis 4:* Organizational identification mediates the positive relationship between LMX and organizational citizenship behavior.
*Hypothesis 5:* Organizational identification mediates the negative relationship between LMX and counterproductive work behavior.

## Overview of Studies

We tested our hypotheses across two field and two experimental studies in an effort to replicate and triangulate our results, employing complementary research designs and sampling strategies (for in-depth discussions, see Turner et al., 2017; Aguinis et al., 2019; Podsakoff and Podsakoff, 2019). In study 1, we employed an initial cross-sectional field study. Because cross-sectional designs are considered to be a basic tool for conducting research that has certain methodological draw-backs by design (e.g., Conway and Lance, 2010; Podsakoff et al., 2012; Spector, 2019), we used a time-lagged field research design in study 2. To further strengthen the validity and the generalizability of our findings, we conducted two online experiments sampling employees from the United States using the crowdsourcing platform MTurk (e.g., Buhrmester et al., 2011, 2018; Porter et al., 2019). Specifically, to corroborate our pattern of results experimentally (e.g., Shadish et al., 2002; Podsakoff and Podsakoff, 2019; Spector, 2019), in study 3, we conducted a recall experiment [relatedly, see Yam et al. (2017)], and in study 4, we employed a vignette experiment (Aguinis and Bradley, 2014). Because the procedures of the respective field and experimental studies differed only slightly, we jointly describe our general procedures and only distinguish between the studies when needed, respectively.

## Field Studies

### Method

#### Participants and Procedure

In study 1, we employed cross-sectional survey and student-recruited sampling (Wheeler et al., 2014) to collect self-report data on 203 employees in Switzerland. Because 15 participants indicated themselves as self-employed, we excluded them from further data analysis. Thus, we based our data analysis on the final sample of 188 employees. In study 2, we employed a prospective two-wave survey design, implementing a lag of 1 month to mitigate a potential common method bias (Podsakoff et al., 2012). Using student-recruited sampling again (Wheeler et al., 2014), 614 employees started to fill in our survey. At time 1, 583 participants completed the first survey, and at time 2, 1 month later, 502 answered our questions regarding the discretionary behaviors, namely, CWB and OCB. No participant out of these 502 indicated herself or himself as self-employed, and, thus, the final sample consisted of 502 employees.

#### Measures and Covariates

We collected the data online using SoSci Survey (Version 3.1.04; Leiner, 2019) and, if not mentioned otherwise, translated all scales into German using the back-translation procedure as recommended by Brislin (1970). Furthermore, if not mentioned otherwise, we measured all items on visual analog scales (0 = “strongly disagree” to 100 = “strongly agree”) because they display superior measurement qualities in comparison to traditional Likert-type response scales and, ultimately, provide data on an interval scale (e.g., Reips and Funke, 2008; Rausch and Zehetleitner, 2014; Kuhlmann et al., 2017).

We described both studies to potential participants as psychological research investigating attitudes and behaviors at the workplace covering different aspects of a typical workday. Welcoming the participants to the actual survey, we assured them of anonymity as well as of data security due to exclusive storage on an encrypted server to eventually foster more truthful responses (e.g., Tourangeau and Yan, 2007; Anseel et al., 2010; Dalal and Hakel, 2016). Next, the participants were asked page by page to answer the items regarding (1) demographic characteristics, (2) LMX, (3) OI, and (4) CWB and OCB. Within the respective scales, the items were presented in a random order to attenuate potential response biases, such as order, primacy, or recency bias (Saris and Gallhofer, 2014).

##### Leader–member exchange

We measured LMX using Schyns’ (2002) validated German version of the LMX-7 scale by Graen and Uhl-Bien (1995), which consists of seven items. We slightly adapted the items to fit the format of our standardized response format (e.g., “My supervisor understands my job-related problems and needs”).

##### Organizational identification

In study 1, we operationalized two components of OI by using (1) the six-item scale by Mael and Ashforth (1992) to assess OI’s cognitive component in terms of perceived oneness with the organization (e.g., “When I talk about my organization, I usually say ‘we’ rather than ‘they”’) and (2) the five-item scale by Blader and Tyler (2009) to assess OI’s affective component in terms of pride in the organizational membership (e.g., “I am proud to tell others where I work”). In study 2, we additionally used the five-item scale by Blader and Tyler (2009) to assess OI’s evaluative component in terms of respect from organizational members for being an organizational member.

##### Counterproductive work behavior

Following the methodological recommendations regarding the measurement of CWB and OCB (Dalal, 2005), we measured CWB using the CWB-C scale by Spector et al. (2010), which consists of 10 behavioral items (e.g., “I came to work late without permission”). In doing so, we accommodated meta-analytic findings that there is one general latent factor comprising CWB (e.g., Berry et al., 2007; Marcus et al., 2016). Specifically, we asked the participants to judge how often they had shown the respective behaviors at work (1) over the last 6 months in study 1 and (2) over the last month in study 2 (0 = “never” to 100 = “daily”).

##### Organizational citizenship behavior

Relatedly, we employed the OCB-C scale by Spector et al. (2010), which consists of 10 items to measure OCB (e.g., “I offered suggestions to improve how work is done”). In doing so, we acknowledged that research has consolidated to focus on OCB as a whole instead of overemphasizing its potential sub-dimensions (e.g., Spector and Fox, 2010; Spector and Che, 2014; Spitzmüller et al., 2018). We asked the participants again to indicate the frequency of engaging in the respective behavior at work (1) over the last 6 months in study 1 and (2) over the last month in study 2 (0 = “never” to 100 = “daily”).

##### Demographic characteristics

We collected the participants’ gender, age, organizational tenure, and employment status (self-employed or employed).

#### Analytic Strategy

To test our theoretical model in a comprehensive and rigorous manner, we applied latent variable modeling [i.e., confirmatory factory analyses (CFA) and structural equation modeling (SEM)]. This analytic approach explicitly allows (1) to inspect the fit of a specified model to the actual data, (2) to correct for measurement error, and (3) to compare alternative models (e.g., Cole and Preacher, 2014; Brown, 2015; Kline, 2016). First, we specified several competing CFA models to establish a well-fitting measurement model. Second and against the background of a well-fitting measurement model, we applied SEM to test our hypotheses. Importantly, no residuals were allowed to covary in any model because there was no theoretical rationale to do so (e.g., Landis et al., 2009; Kline, 2016; Pan et al., 2017). We evaluated acceptable model fit in light of five fit indices: (1) absolute test of fit, χ^2^, (2) comparative fit index (CFI) ≥ 0.90, (3) Tucker–Lewis index (TLI) ≥ 0.90, (4) root mean square error of approximation (RMSEA) ≤ 0.05, and (5) standardized root mean square residual (SRMR) ≤ 0.08 (Hu and Bentler, 1999).

We conducted all statistical analyses using the statistical environment R (Version 3.4.0; R Development Core Team, 2020) and particularly used the packages *lavaan* (Version 0.6-1.1141; Rosseel, 2012) and *RMediation* (Version 1.1.4; Tofighi and MacKinnon, 2011). To determine the proper estimator, we assessed the assumptions of the *maximum likelihood* estimator: (1) Because we used visual analog scales, the assumption of measurement on an interval-scale level can be considered as fulfilled (e.g., Reips and Funke, 2008; Rausch and Zehetleitner, 2014; Kuhlmann et al., 2017); (2) Furthermore, we tested the respective data for multivariate normality using the Henze–Zirkler test (Henze and Zirkler, 1990), which is provided in the *MVN* package (Version 5.7; Korkmaz et al., 2014).

### Results

Tables 1, 2 display the descriptive statistics, zero-order correlations, as well as the internal consistencies for study 1 and study 2. The data of both studies were not distributed in a multivariate normal manner—thus, we used the robust maximum likelihood estimator to obtain robust standard errors and a corrected test statistic to evaluate model fit (Yuan and Bentler, 1998).

**TABLE 1:** Zero-order correlations, internal consistencies, and descriptive statistics for study 1.

	*M*	*SD*	1	2	3	4	5	6	7	8
(1) Gender (1 = ♂)	102 ♀, 86 ♂									
(2) Age	34.39	12.03	–0.12							
(3) Tenure	5.54	7.00	–0.09	0.65***						
(4) LMX	57.96	25.43	–0.09	–0.12	–0.09	(0.94)				
(5) OI cognitive	57.09	22.08	–0.09	0.14	–0.02	0.33***	(0.84)			
(6) OI affective	70.47	19.31	0.04	0.26***	0.12	0.35***	0.45***	(0.81)		
(7) OCB	66.39	17.11	–0.06	0.29***	0.20**	0.06	0.31***	0.25***	(0.86)	
(8) CWB	12.51	11.79	–0.11	−0.23**	–0.11	−0.20**	−0.16*	−0.42***	0.02	(0.82)

N = 188. Standardized Cronbach’s alpha coefficients are reported along the diagonal in parentheses. LMX, leader–member exchange; OI cognitive, cognitive component of organizational identification; OI affective, affective component of organizational identification; OCB, organizational citizenship behavior; CWB, counterproductive work behavior. *p < 0.05, **p < 0.01, ***p < 0.001.

**TABLE 2:** Zero-order correlations, internal consistencies, and descriptive statistics for study 2.

Variable	*M*	*SD*	1	2	3	4	5	6	7	8	9
(1) Gender (1 = ♂)	185 ♀, 317 ♂
(2) Age	30.66	9.50	–0.06								
(3) Tenure	2.55	4.88	–0.07	0.52***							
(4) LMX	70.12	21.02	–0.03	–0.04	0.05	(0.92)					
(5) OI cognitive	53.37	23.17	−0.12**	0.08	0.12**	0.40***	(0.86)				
(6) OI affective	63.88	21.52	–0.08	–0.02	0.03	0.42***	0.59***	(0.84)			
(7) OI evaluative	64.17	20.85	−0.12**	0.01	0.10*	0.64***	0.57***	0.60***	(0.87)		
(8) OCB	53.17	18.58	–0.08	0.10*	0.13**	0.29***	0.36***	0.33***	0.40***	(0.85)	
(9) CWB	8.65	9.06	−0.14**	−0.11*	0.01	−0.15**	–0.08	−0.17***	−0.19***	0.03	(0.77)

N = 502. Standardized Cronbach’s alpha coefficients are reported along the diagonal in parentheses. LMX, leader–member exchange; OI cognitive, cognitive component of organizational identification; OI affective, affective component of organizational identification; OI evaluative, evaluative component of organizational identification; OCB, organizational citizenship behavior; CWB, counterproductive work behavior. *p < 0.05, **p < 0.01, ***p < 0.001.

Table 3 displays the results of the CFAs for both studies, namely, (1) a one-factor model in which we specified all items to load onto one factor, (2) a three-factor model in which we specified LMX, both components of OI, and CWB and OCB to form one factor, respectively, (3) a five-factor model in which we specified LMX, the specific facets of OI, CWB, and OCB to load onto one factor, respectively, (4) an adapted version of model 3 in which we specified OI as a second-order factor to subsume both components, and (5) a parceled version of model 4 to reduce model complexity (e.g., Landis et al., 2000; Brown, 2015; Kline, 2016). Specifically, we created three indicator parcels for each construct by adapting the *item-to-construct balance* principle in model 5 (e.g., Little et al., 2002; Williams and O’Boyle, 2008; Brown, 2015). Overall, model 5 suggested acceptable fit to and thus a valid representation of the data in both studies: study 1: χ^2^(82) = 103.29, *p* = 0.06, CFI = 0.99, TLI = 0.98, RMSEA = 0.04 (90% CI: 0.00–0.06, *p* = 0.85), SRMR = 0.05; study 2: χ^2^(126) = 522.50, *p* < 0.001, CFI = 0.93, TLI = 0.91, RMSEA = 0.08 (90% CI: 0.07–0.09, <0.001), SRMR = 0.07.

**TABLE 3:** Confirmatory factor analyses for studies 1 and 2.

Model	χ^2^	*df*	*P*	CFI	TLI	RMSEA (90% CI, *p*)	SRMR
**Study 1**							
Model 1	2,620.41	665	<0.001	0.37	0.34	0.13 (0.13–0.14, <0.001)	0.16
Model 2	1,798.79	662	<0.001	0.64	0.62	0.10 (0.10–0.11, <0.001)	0.13
Model 3	1,295.15	655	<0.001	0.80	0.79	0.08 (0.07–0.08, <0.001)	0.08
Model 4	1,305.38	657	<0.001	0.80	0.78	0.08 (0.07–0.08, 0.07)	0.08
Model 5	103.29	82	0.06	0.99	0.98	0.04 (0.00–0.06, 0.85)	0.05
**Study 2**							
Model 1	5,085.07	860	<0.001	0.52	0.50	0.11 (0.10–0.11, <0.001)	0.11
Model 2	3,627.31	857	<0.001	0.69	0.67	0.09 (0.08–0.09, <0.001)	0.11
Model 3	2,205.55	845	<0.001	0.85	0.84	0.06 (0.06–0.06, <0.001)	0.08
Model 4	2,260.21	851	<0.001	0.85	0.84	0.06 (0.06–0.06, <0.001)	0.08
Model 5	522.50	126	<0.001	0.93	0.91	0.08 (0.07–0.09, <0.001)	0.07

*Study 1: N = 188; study 2: N = 502.*

**FIGURE 2:**
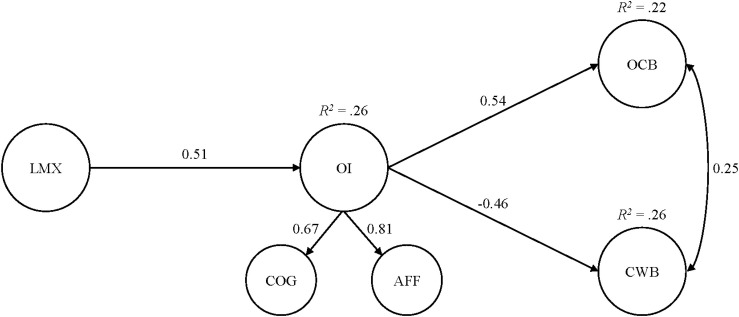
Results from the full structural equation model for study 1. Only the statistically significant coefficients, from the full structural equation model, that stem from the completely standardized solution are displayed. *N* = 188. LMX, leader–member exchange; OI, organizational identification; COG, cognitive component of organizational identification; AFF, affective component of organizational identification; OCB, organizational citizenship behavior; CWB, counterproductive work behavior.

**FIGURE 3:**
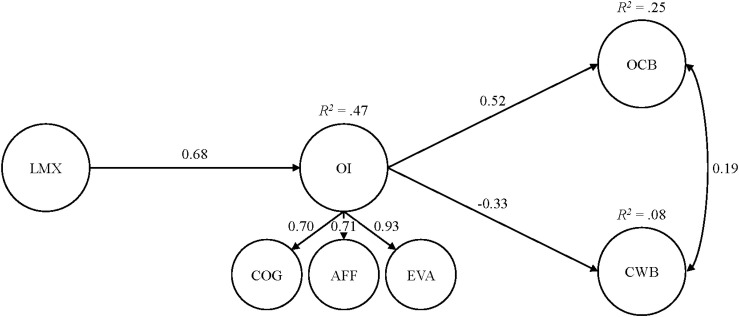
Results from the full structural equation model for study 2. Only the statistically significant coefficients, from the full structural equation model, that stem from the completely standardized solution are displayed. *N* = 502. LMX, leader–member exchange; OI, organizational identification; COG, cognitive component of organizational identification; AFF, affective component of organizational identification; EVA, evaluative component of organizational identification; OCB, organizational citizenship behavior; CWB, counterproductive work behavior.

On the basis of model 5, in both studies, we applied SEM and regressed (1) OI onto LMX and (2) CWB and OCB onto OI and onto LMX, respectively, to allow the estimation of all potentially relevant direct as well as indirect effects. In both studies, the resulting models displayed good fit to the data: study 1: χ^2^(82) = 103.27, *p* = 0.06, CFI = 0.99, TLI = 0.98, RMSEA = 0.04 (90% CI: 0.00–0.06, *p* = 0.86), SRMR = 0.05; study 2: χ^2^(126) = 522.40, *p* < 0.001, CFI = 0.93, TLI = 0.91, RMSEA = 0.08 (90% CI:0.08–0.09, <0.001), SRMR = 0.07 (Table 4 and Figures 2, 3). In turn, we found statistically significant positive associations of LMX with OI [study 1: *b*^∗^ = 0.51 (95% CI: 0.33–0.67); study 2: *b*^∗^ = 0.68 (95% CI: 0.60–0.77)] but no direct effects of LMX regarding both CWB and OCB. In addition, OI was statistically significantly related to CWB in a negative way [study 1: *b*^∗^ = −0.46 (95% CI: −0.67–−0.24); study 2: *b*^∗^ = −0.33 (95% CI: −0.54–−0.11)] and to OCB in a positive way [study 1: *b*^∗^ = 0.54 (95% CI: 0.24–0.84); study 2: *b*^∗^ = 0.52 (95% CI: 0.37–0.66)]. Interestingly, in both studies, the residual correlations between the endogenous constructs, CWB and OCB, were statistically significant and positive. Finally, following the recommendations by Tofighi and MacKinnon (2011), we applied the distribution-of-the-product method for building 95% confidence intervals for the standardized indirect effects. In both studies, we found (1) statistically significant negative indirect effects of LMX *via* OI onto CWB [study 1: *b*^∗^ = −0.23 (95% CI: −0.38–−0.11); study 2: *b*^∗^ = −0.22 (95% CI: −0.38–−0.07)] and (2) statistically significant positive indirect effects of LMX *via* OI onto OCB [study 1: *b*^∗^ = 0.27 (95% CI: 0.11–0.47); study 2: *b*^∗^ = 0.35, *SE* = 0.06, 95% CI: (0.25–0.46); Table 4]. Following Becker (2005), we ran all of our analyses with and without demographic controls, and the results were essentially identical with the inclusion of these variables. In sum, we found empirical support for all our postulated hypotheses.

**TABLE 4:** Full structural equation models for studies 1 and 2.

Path	Study 1					Study 2				
	95% CI					95% CI				
	b*	*SE*	Lower	Upper	*p*	b*	*SE*	Lower	Upper	*p*
OI on										
LMX	0.51	0.09	0.33	0.67	<0.001	0.68	0.04	0.60	0.77	<0.001
CWB on										
OI	−0.46	0.11	−0.67	−0.24	<0.001	−0.33	0.11	−0.54	−0.11	<0.01
LMX	−0.03	0.11	−0.24	0.18	0.79	0.07	0.09	−0.10	0.23	0.44
OCB on										
OI	0.54	0.15	0.24	0.84	<0.001	0.52	0.07	0.37	0.66	<0.001
LMX	−0.20	0.12	−0.44	0.04	0.10	−0.02	0.08	−0.18	0.13	0.76
CWB with										
OCB	0.25	0.12	0.02	0.47	<0.05	0.19	0.05	0.10	0.29	<0.001
LMX → OI → CWB	−0.23	0.07	−0.38	−0.11	–	−0.22	0.08	−0.38	−0.07	–
LMX → OI → OCB	0.27	0.09	0.11	0.47	–	0.35	0.06	0.25	0.46	–

*Study 1: N = 188; study 2: N = 502. Path coefficients stem from the completely standardized solution of the full structural equation model. OI, organizational identification; LMX, leader–member exchange; CWB, counterproductive work behavior; OCB, organizational citizenship behavior.*

### Discussion

Across both field studies, we found a consistent pattern of results that supports our theoretical model. In particular and conditional upon the data, LMX is positively associated with OI, which, in turn, is negatively associated with CWB and positively with OCB. In addition, we did not find direct effects of LMX onto OCB or CWB, but, as hypothesized, we found the respective indirect effects. As such, this consistent pattern of results lends initial support to a leader’s pivotal role in strengthening an employee’s OI (e.g., Carmeli et al., 2011; Loi et al., 2014; Zhao et al., 2019). In turn, OI appears to curb negative and, at the same time, foster positive discretionary behaviors at work. As such, employees who are strongly identified with their organization are more inclined to refrain from violating organizational norms by showing CWB and, even more so, to exceed organizational norms by displaying OCB. In other words, OI appears to serve as a unique factor affecting both positive and negative voluntary behaviors at the workplace [relatedly, see Hunt (1996)].

Although we addressed certain methodological limitations of study 1 by employing a time-lagged study design in study 2 (i.e., mitigation of common methods bias; Podsakoff et al., 2012), the overall research design hampers rigorous causal inferences (e.g., Spencer et al., 2005; Shadish et al., 2002; Pirlott and MacKinnon, 2016). Specifically, all postulated associations are eventually assumed to be causal—yet, these claims cannot be empirically corroborated by the non-experimental study designs that we employed in the field (Shadish et al., 2002). Consequently, to test our theoretical model more rigorously, we chose to employ a randomized experimental design because “when mediation models are tested by randomized experimental means, inferences about mediation rest on a very firm foundation” (Stone-Romero and Rosopa, 2008, p. 330). Specifically, we conducted two experimental studies in which we manipulated LMX using a recall task [study 3; relatedly, see Yam et al. (2017)] and a vignette task (study 4; Aguinis and Bradley, 2014).

## Experimental Studies

### Method

#### Design and Procedure

To conduct our two online experiments manipulating LMX, we sampled from the crowdsourcing platform MTurk. To assure a high data quality, we took several preventive measures following recent methodological recommendations: First, we randomly spread four *bogus* items across the experimental materials, included an initial warning for the participants that some items might strike them as odd, and recorded the time to complete the experiment in seconds to check for potential careless responders (i.e., insufficient effort responding; e.g., De Simone et al., 2015; Huang et al., 2015; De Simone and Harms, 2018). Second, we set a 97% approval rate as a qualification criterion for potential MTurk workers to be included in the study (Peer et al., 2014). Third, to decrease dropout rates in light of the experimental manipulation, we informed the participants upfront that they might come across a task where they need to type a few sentences and appealed to the participants’ conscience by telling them that our research depends on good data quality (Zhou and Fishbach, 2016). Finally, we offered the participants $2 for their complete participation which, in light of the average completion time of roughly 10 min, resulted in an hourly wage of approximately $12 (Gleibs, 2017).

Resembling our field studies, we described our research to potential participants as investigating attitudes and behaviors at the workplace with a specific focus on different aspects of a typical workday. Upon initial participation, we assessed the participants’ demographic characteristics and then randomly assigned them into one of three conditions. In study 3, the recall experiment, the participants were asked to type in three to five sentences describing situations depicting (1) a high-quality relationship with their supervisor (high-LMX condition), (2) a low-quality relationship with their supervisor (low-LMX condition), or (3) particular activities that they usually pursue in their spare time (control condition). In study 4, the vignette experiment, we randomly assigned the participants into one of three vignette conditions, where the participants were asked to imagine either (1) a high-quality relationship with an imaginary supervisor (high-LMX condition), (2) a low-quality relationship with an imaginary supervisor (low-LMX condition), or (3) certain hobbies that they like to pursue in their spare time (control condition). In both studies, initially, the participants in the LMX conditions read a few introductory sentences about different characteristics of relationship quality between supervisors and employees (high-LMX and low-LMX condition) or about spare time being an important aspect of one’s life besides work (control condition). In each LMX condition, we also provided two respective examples based on items of the multidimensional measure of LMX (LMX-MDM; Liden and Maslyn, 1998). Then, we randomly assigned the participants to one of three conditions to manipulate LMX.

In study 3, we manipulated LMX *via* recall task because this method has been successfully employed in other psychological experimental studies (e.g., Yam et al., 2017). Specifically, we asked the participants to recall (1) particular situations depicting a high-quality relationship with their supervisor based on mutual trust, respect, liking, and/or reciprocal influence (high-LMX condition), (2) particular situations depicting a low-quality relationship with their supervisor lacking in mutual trust, respect, liking, and/or reciprocal influence (low-LMX condition), or (3) particular activities that they liked to pursue in their spare time (control condition). Having read the introductory sentences, the participants were asked to type in three to five sentences describing situations in accordance with the respective condition. Exemplary for the respective participants’ responses, situations such as the following were described: (1) high-LMX: “My supervisor helped me complete quality assurance logs because we were very busy and I could not finish in time,” (2) low-LMX: “My supervisor questioned where I was when I was in a meeting and not in the office,” or (3) control: “I run outside to improve my health and unwind.”

In study 4, we manipulated LMX *via* a vignette task and, in doing so, followed the methodological recommendations by Aguinis and Bradley (2014). Specifically, we phrased our vignettes for the high-LMX and the low-LMX conditions in close resemblance to the LMX-MDM scale by Liden and Maslyn (1998). We explicitly chose this scale as a reference to attenuate potential verbatim carry-over effects that might occur from phrasing vignettes along the lines of the same scale that we employed to assess LMX (i.e., LMX-7; Graen and Uhl-Bien, 1995) for the respective manipulation check. Although both measures of LMX somewhat differ conceptually and verbally, meta-analytic evidence suggests a strong correlation between the LMX-MDM and the LMX-7 scales (Martin et al., 2016). In addition, we made sure that the participants across all conditions had roughly the same amount of overall information and the exact same information regarding the organization and the tenure they supposedly had spent with their imaginary supervisor. In turn, the participants were asked to read one of the following vignettes and imagine themselves in one of the following scenarios:

*You work for a mid-size organization in the private sector. You have been working under your present supervisor for about 2 years now and you (dis)like working with this supervisor. You do (not) respect your supervisor*’*s knowledge of and competence on the job and you do (not) particularly value his/her opinion. Also, you do (not) like your supervisor very much as a person. (Un)fortunately, you can(not) always count on the supervisor to defend you in times of crises. He/She is the kind of supervisor who would (not) defend your work actions to a superior without complete knowledge of the issue in question. In return, you (refrain from) do(ing) work for your supervisor that goes beyond what is specified in your job description.* [The differences between the high- and the low-LMX conditions are printed in parentheses.]

For the control condition, we asked the participants to imagine themselves in the following situation while leaving out any details regarding a potential supervisor:

You work for a mid-size organization in the private sector. In your spare time on weekdays, you like to do relaxing activities. Usually, you read a book or watch a movie. Sometimes you go to the movies nearby with friends. You also like exercising and cooking. You enjoy trying out new recipes. At the weekend, you go out quite often and meet up with friends and family, but as you also like being outdoors, you spend some weekends hiking in nature. You enjoy the mountains and the fresh air. Sometimes a friend joins you on your trip.

#### Participants

We conducted our two online experiments on the crowdsourcing platform MTurk. In doing so, we recruited 172 full-time employed adults in study 3 and 207 full-time employed adults in study 4.

#### Measures and Covariates

We designed the online experiments to closely resemble our field studies. Thus, we provided the materials in English using SoSci Survey (Version 3.1.04; Leiner, 2019) and measured the variables of interest using VAS (e.g., Reips and Funke, 2008; Rausch and Zehetleitner, 2014; Kuhlmann et al., 2017), ranging from 0 to 100 with the verbal anchors “strongly disagree” and “strongly agree” as response scales, if not mentioned otherwise.

##### Organizational identification

We measured the three components of OI, namely, (1) perceived oneness with the organization (six items; Mael and Ashforth, 1992), (2) pride in the organizational membership (five items; Blader and Tyler, 2009), and (3) respect from organizational members for being an organizational member (five items; Blader and Tyler, 2009).

##### CWB and OCB

We measured CWB using the 10-item CWB-C scale by Spector et al. (2010) and measured OCB using the 10-item OCB-C scale by Spector et al. (2010). Specifically, we asked the participants to indicate the likelihood of engaging in each of the presented behaviors over the next 6 months at work, respectively (0 = “never” to 100 = “daily”).

##### Insufficient effort responding

To flag the participants who are potentially responding carelessly to our measures, we randomly spread the four items with the highest loadings from the insufficient effort responding (IER) scale by Huang et al. (2015) over the entire survey (e.g., “I can teleport across time and space”). In addition, we recorded the total completion time in seconds (e.g., Huang et al., 2012; De Simone et al., 2015; De Simone and Harms, 2018).

##### Demographic characteristics

We asked the participants to indicate their (1) gender, (2) age in years, (3) tenure with the current organization in years, and (4) whether they currently had a supervisor. The participants who reported to currently not have a supervisor were subsequently thanked for their interest in our study but immediately excluded from further participation in it.

#### Manipulation Check

To check whether the manipulation of LMX *via* the recall task in study 3 and *via* the vignette task in study 4 had worked, we asked the participants in both studies to answer the seven items of the LMX-7 scale by Graen and Uhl-Bien (1995) with regard to how they viewed the relationship with their supervisor, keeping in mind the situations or the scenarios they had just described or read about, respectively.

### Results

We again conducted all statistical analyses in R (Version 3.4.0; R Development Core Team, 2020). Following the recommendations by De Simone et al. (2015) and De Simone and Harms (2018), we first screened the data by applying (1) a direct criterion based on the IER scale to identify the participants who were responding carelessly and (2) an archival criterion based on the participants’ response time regarding the entire online experiment to identify the participants that were responding too quickly. Regarding study 3, we excluded 24 out of the initial 172 participants due to an average IER score above 10 and 17 participants due to an average response time of less than 2 s per item (Huang et al., 2012); this resulted in a final sample of 131 participants for study 3. Resembling these criteria in study 4, we excluded 28 out of the 207 participants due to suspected careless responding and 40 participants due to a particularly low average response. In turn, the final sample of study 4 comprised 139 participants (see Appendix for zero-order correlations, internal consistencies, and descriptive statistics for studies 3 and 4).

Next, we conducted one-way ANOVAs to check whether the manipulation of LMX worked. In study 3, there was a statistically significant main effect of the experimental manipulation on LMX, *F*(2,128) = 5.83, *p* < 0.01, $\eta_{\text{p}}^{2}$ = 0.08. A *post hoc* comparison of the experimental conditions using Tukey HSD test revealed statistically significant differences between the high-LMX (*M* = 81.34, *SD* = 18.29) and the low-LMX conditions (*p* < 0.01) and between the low-LMX (*M* = 68.21, *SD* = 19.95) and the control conditions (*M* = 77.78, *SD* = 16.13) (*p* < 0.05), but not a statistically significant difference between the high-LMX and the control conditions (*p* = 0.61). Because the difference between the high- and the low-LMX conditions was statistically significant, we considered the overall manipulation of LMX to be successful in study 3. With regard to study 4, we found a statistically significant main effect of the experimental condition regarding LMX, *F*(2,136) = 211.10, *p* < 0.001, $\eta_{\text{p}}^{2}$ = 0.76. *Post hoc* comparisons using Tukey HSD test indicated statistically significant (*p* < 0.001) differences between all three conditions, specifically (1) the high-LMX (*M* = 89.91, *SD* = 7.51), (2) the low-LMX (*M* = 22.63, *SD* = 21.69), and (3) the control conditions (*M* = 75.92, *SD* = 16.56). In turn, we considered the manipulation of LMX *via* vignette as successful.

To test our hypotheses, we conducted path analyses focusing on direct as well as indirect effects using the R package *lavaan* (Version 0.6-1.1141; Rosseel, 2012). Because the data did not follow a multivariate normal distribution and because our sample sizes were rather small, we used the robust maximum likelihood estimator to obtain robust standard errors (Yuan and Bentler, 1998). We specified models in which the paths from LMX to OI, OCB, and CWB, direct effects from OI to CWB and OCB, as well as a covariance between these two endogenous constructs were estimated. Importantly, we estimated the path analytic models for the full samples (i.e., all three conditions) and for the manipulated sample (i.e., low-LMX and high-LMX conditions).

Both studies yielded a consistent pattern of findings in that LMX was positively associated with OI, which, in turn, was negatively associated with CWB and positively associated with OCB. Furthermore, there were no statistically significant direct effects of LMX on either CWB or OCB (Tables 5, 6). In addition, the correlation between CWB and OCB was not statistically significant in any of the estimated models. Consequently, using the R package *RMediation* (Version 1.1.4; Tofighi and MacKinnon, 2011), we applied the distribution-of-product method for building 95% confidence intervals for the standardized indirect effects. Analyzing the full sample of study 3, we found the two indirect effects to be statistically significant because the respective confidence intervals excluded zero: (1) LMX *via* OI onto CWB, *b*^∗^ = −0.19, *SE* = 0.05, 95% CI: (−0.30–−0.09) and (2) LMX *via* OI onto OCB, *b*^∗^ = 0.31, *SE* = 0.06, 95% CI: (0.20–0.44). Analyzing only the participants in the high- and the low-LMX conditions, thereby excluding the control condition, essentially yielded the same pattern of results in study 3: (1) LMX *via* OI onto CWB, *b*^∗^ = −0.20, *SE* = 0.07, 95% CI: (−0.35–−0.06) and (2) LMX *via* OI onto OCB, *b*^∗^ = 0.39, *SE* = 0.09, 95% CI: (0.24–0.56). Analyzing the complete sample of study 4, we found the following statistically significant indirect effects: (1) LMX *via* OI onto CWB, *b*^∗^ = −0.25, *SE* = 0.09, 95% CI: (−0.43–−0.07) and (2) LMX *via* OI onto OCB, *b*^∗^ = 0.57, *SE* = 0.07, 95% CI: (0.44–0.70) (Table 6). Analyzing only the participants in the high- and the low-LMX conditions again, thereby excluding the control condition, we again found the following indirect effects to be statistically significant in study 4: (1) LMX *via* OI onto OCB, *b*^∗^ = 0.60, *SE* = 0.07, 95% CI: (0.47–0.74) and (2) LMX *via* OI onto CWB, *b*^∗^ = −0.28, *SE* = 0.11, 95% CI: (−0.50–−0.06). Overall, the consistent pattern of results across both experimental studies yielded further empirical support for our theoretical model.

**TABLE 5:** Path analyses for study 3.

Path	Recall (including control condition)					Recall (excluding control condition)				
	95% CI					95% CI				
	b*	*SE*	Lower	Upper	*p*	b*	*SE*	Lower	Upper	*p*
OI on										
LMX	0.49	0.08	0.35	0.64	<0.001	0.60	0.08	0.43	0.77	<0.001
CWB on										
OI	−0.39	0.09	−0.57	−0.22	<0.001	−0.33	0.11	−0.55	−0.10	<0.01
LMX	−0.01	0.11	−0.23	0.21	0.93	0.07	0.13	−0.20	0.33	0.63
OCB on										
OI	0.65	0.08	0.49	0.81	<0.001	0.65	0.10	0.44	0.85	<0.001
LMX	0.08	0.08	−0.08	0.24	0.32	0.09	0.11	−0.12	0.30	0.40
CWB with										
OCB	−0.05	0.09	−0.23	0.13	0.57	−0.10	0.09	−0.28	0.08	0.29
LMX–OI–CWB	−0.19	0.05	−0.30	−0.09	–	−0.20	0.08	−0.35	0.06	–
LMX–OI–OCB	0.32	0.06	0.24	0.56	–	0.39	0.09	0.24	0.56	–

*Recall (including control condition): N = 131; recall (excluding control condition): n = 83. Path coefficients stem from the completely standardized solution. OI, organizational identification; LMX, leader–member exchange; CWB, counterproductive work behavior; OCB, organizational citizenship behavior.*

**TABLE 6:** Path analyses for study 4.

Path	Vignette (including control condition)					Vignette (excluding control condition)				
	95% CI					95% CI				
	b*	*SE*	Lower	Upper	*p*	b*	*SE*	Lower	Upper	*p*
OI on										
LMX	0.79	0.04	0.71	0.87	<0.001	0.79	0.05	0.71	0.88	<0.001
CWB on										
OI	−0.31	0.12	−0.54	−0.09	<0.01	−0.36	0.14	−0.63	−0.08	<0.05
LMX	−0.20	0.11	−0.42	0.02	0.07	−0.13	0.13	−0.38	0.13	0.33
OCB on										
OI	0.72	0.07	0.58	0.87	<0.001	0.76	0.08	0.61	0.91	<0.001
LMX	0.15	0.08	−0.001	0.30	0.05	0.14	0.08	−0.01	0.30	0.07
CWB with										
OCB	−0.12	0.10	−0.32	0.09	0.26	−0.16	0.13	−0.40	0.09	0.21
LMX–OI–CWB	−0.25	0.09	−0.43	−0.07	–	−0.28	0.11	−0.51	−0.06	–
LMX–OI–OCB	0.57	0.07	0.44	0.70	–	0.60	0.07	0.47	0.74	–

*Vignette (including control condition): N = 139; vignette (excluding control condition): n = 91. Path coefficients stem from the completely standardized solution. OI, organizational identification; LMX, leader–member exchange; CWB, counterproductive work behavior; OCB, organizational citizenship behavior.*

### Discussion

Closely resembling the field studies, we found consistent results in both experimental studies which corroborate our theoretical model. In particular and conditional upon the data, LMX is statistically significantly associated with OI, which, in turn, is negatively related to CWB and positively related to OCB. Consistent with our theoretical model again, we found indirect effects of LMX *via* OI regarding the discretionary behaviors CWB and OCB. Importantly, this pattern of results was consistent across two different manipulations, namely, a recall task and a vignette task. Overall, these experimental studies provide further support to our notion that OI is a central mechanism linking LMX to discretionary workplace behaviors.

A potential drawback of our experimental vignette study might lie in the fact that the high-LMX and the control conditions did not differ significantly with respect to the manipulation check. Although the participants in the low-LMX condition rated their LMX significantly lower than in the high-LMX condition, the actual mean (*M* = 68.21) was still on the positive side of the response scale (i.e., above 50). Yet, in light of the fact that the high- and the low-LMX conditions significantly differed, we consider our pattern of findings as somewhat robust.

## General Discussion

Across two field and two experimental studies, we found that the quality of employees’ relationship with their direct supervisor (i.e., LMX) positively predicted the extent to which employees identify with their organization, which, in turn, curbed behavior harmful to the organization (i.e., CWB) and fostered desirable behavior in the workplace (i.e., OCB). In all four studies, we identified OI as a pivotal mechanism that can explain why LMX affects discretionary workplace behaviors. As such, we contend that our research, at least partially, answers the respective call by Martin et al. (2016) to study “theory-guided mechanisms that explain the link between LMX and the various dimensions of performance” (p. 104). Furthermore, the empirical support that we provided for the position of OI as a central antecedent of both CWB and OCB directly answers respective calls by Lee et al. (2015, p. 1,062) to “explore organizational identification’s implications for those undesirable behaviors at work.”

### Theoretical Implications

Our findings contribute to the existing literature in several ways. We extend the literature regarding the effects of LMX by having theoretically proposed and empirically illustrated OI as an intervening mechanism that transmits the effects of LMX regarding discretionary behaviors, namely, CWB and OCB. Essentially different from work attitude constructs, such as affective commitment (e.g., Van Knippenberg and Sleebos, 2006; Klein et al., 2012; Lee et al., 2015), OI directly refers to an individual’s identification in terms of the organization and, thus, its norms. Our findings suggest that employees generalize the relationship with their supervisor to the organization as a whole, which leads them to define themselves in terms of the organization and to act according to organizational norms and interests or even exceed them (i.e., refraining from CWB, engaging in OCB).

Besides proposing OI as a mechanism linking LMX and discretionary behavior, we theoretically and empirically illustrated that OI itself plays a pivotal role regarding the emergence of employees’ OCB and, importantly, the deterrence of employees’ CWB. In line with the theoretical propositions by the SIA (Tajfel and Turner, 1986; Turner et al., 1987; Haslam, 2004) in general, our findings support and extend the meta-analytic findings regarding the fundamental role OI appears to play in organizational behavior in general (e.g., Riketta, 2005; Riketta and Van Dick, 2005; Lee et al., 2015). In particular, strongly identified employees appear to choose desirable ways of deviating from organizational norms (i.e., OCB) and to refrain from undesirable ways of deviating from organizational norms (i.e., CWB; relatedly, see Blanton and Christie, 2003). As such, OI can be considered as a unique factor that oppositely but simultaneously affects both negative discretionary behaviors (i.e., CWB) as well as positive discretionary behaviors [i.e., OCB; for a related discussion, see Hunt (1996)].

However, even if OI truly is somewhat of an almighty engine of organizational behavior, there is also reason to be careful due to its potential negative consequences (e.g., Dukerich et al., 1998; Vadera and Pratt, 2013; Conroy et al., 2017). Specifically, if employees were strongly identified with an organization that held questionable norms from a societal or ethical perspective, employees might engage in behavior that could be viewed as desirable from the perspective of the organization and, at the same time, perceived as detrimental by the overarching society (e.g., unethical pro-organizational behavior; e.g., Umphress et al., 2010; Umphress and Bingham, 2011). To provide future research with a more balanced view of the consequences of OI, it might also be promising to extend our empirical work and to investigate these potential negative outcomes of OI.

Finally, drawing from two theoretical frameworks—the SET (Blau, 1964; Cropanzano and Mitchell, 2005; Cropanzano et al., 2017) and the SIA (Tajfel and Turner, 1986; Turner et al., 1987; Haslam, 2004)—we contribute to a more unified understanding of why employees engage in discretionary workplace behavior by empirically testing an integrative model and corroborating previous findings regarding parts of our conceptual model [relatedly, see Tyler and Blader (2003), Blader and Tyler (2009), and O’Boyle et al. (2011)]. We found indirect effects that were somewhat comparable in size across the four complementary studies despite the different methodological approaches employed. Of course, such a comparison should be made with caution because of the standardization by the respective sample-specific standard deviations which obviously can vary across studies (Cohen et al., 2003).

### Limitations and Avenues for Future Research

As Spector (2019, p. 135) noted, “no single study, no matter what the design, is in itself conclusive, but rather, it is a body of research across many researchers using a variety of methods that allow us to have confidence in conclusions.” Our research also has limitations of which we hope will inspire future research. First, a potential drawback of this research lies in the fact that we measured all variables in a self-report manner. Thus, biases, such as common method (Podsakoff et al., 2012), social desirability (Paulhus, 1984), and/or non-response bias (Greco et al., 2015), might lead to exaggerated or somewhat distorted associations of the constructs under investigation. Yet, concerns regarding common method bias are alleviated to some extent because we conducted observational studies—thereby following the recommendations by Podsakoff et al. (2012)—as well as experimental studies. In addition, current methodological recommendations regarding the measurement of sensible constructs, such as CWB, consider self-report to be a prudent source for measuring this private behavior (e.g., Berry et al., 2012; Dalal and Hakel, 2016; Carpenter et al., 2017). Nonetheless, future research might employ more rigorous research designs with multiple measurement points (e.g., Ployhart and MacKenzie, 2015; Liu et al., 2016; O’Laughlin et al., 2018) and explicit investigations of potential non-response biases (e.g., Greco et al., 2015) to strengthen causal inferences.

Second, we focused on an employee’s perception of the LMX quality at the individual level and, in doing so, did not account for the dyadic nature of LMX or other even higher levels of analysis (e.g., work group; cf. Gooty and Yammarino, 2016; Epitropaki et al., 2018; Martin et al., 2018). Exemplarily, Gooty and Yammarino (2016) investigated the relationship of LMX at the individual, the dyadic, and the group levels and found this multilevel perspective to provide a complex picture of the manifold effects of LMX: While LMX dispersion at the dyadic level attenuated the positive relationship of LMX and performance at the individual level, LMX differentiation at the group level turned out to be dysfunctional for individual performance. Therefore, we consider future research adopting a multilevel perspective—thereby acknowledging contextual factors such as dyadic and work group characteristics—to study the effects of LMX regarding OI and subsequently CWB and OCB a promising avenue [see also Klein et al. (2000), Martin et al. (2018), and Seo et al. (2018)].

Third, we theoretically postulated and empirically demonstrated the effect of LMX regarding OI but did not control for a SET construct referring to the organization, such as perceived organizational support (POS; e.g., Rhoades and Eisenberger, 2002; Eisenberger and Stinglhamber, 2011; Eisenberger et al., 2019). Specifically, Lavelle et al. (2007) argued that employees hold distinct social exchange relationships with multiple organizational foci (e.g., organization, supervisors) and suggested that employees rather reciprocate treatment they experienced within foci than to generalize to others. Although meta-analytic evidence does not provide strong evidence supporting multi-foci arguments (Colquitt et al., 2013), future research might explore the role of organization-focused constructs, such as POS, within our theoretical model, thereby investigating the claim by Gerstner and Day (1997) that “the relationship with one’s supervisors [is] a lens through which the entire work experience is viewed” (p. 840) more rigorously.

Fourth, despite research having somewhat consolidated on considering CWB and OCB to have general underlying respective factors (e.g., Berry et al., 2007; Marcus et al., 2016; Spitzmüller et al., 2018), these discretionary behaviors have numerous behavioral manifestations that might call for a more fine-grained conceptualization and investigation of these constructs. In this current research, we considered both in their most general forms and took into account Dalal’s (2005) methodological recommendations regarding operationalization [e.g., avoidance of antithetical items; see also Dalal and Carpenter (2018)], yet the very definitions of both constructs stress the normative component of the behaviors in that specific reference made to an employee’s organization (e.g., Warren, 2003; Palmer, 2012; Götz et al., 2019). In other words, different behaviors might be viewed as destructively or constructively deviant by different organizations or even different workgroups (e.g., Robinson and Kraatz, 1998; Liao et al., 2004; Bollmann and Krings, 2016). As such, future research might explore deviance within one single organization, thereby explicitly taking into account the specific normative context of the employees under investigation [exemplarily, see Dineen et al. (2006)].

Fifth, against the basis of our studies, we currently cannot rule out potential alternative mechanisms for the association of LMX and CWB as well as OCB (e.g., Spencer et al., 2005; Kline, 2015; Pirlott and MacKinnon, 2016). Thus, we call upon future research to investigate competing intervening mechanisms, such as trust [specifically, see Martin et al. (2016)], to empirically test our claim of OI being a central mediator between LMX and discretionary workplace behaviors. In a similar vein, an interesting addition to our theoretical model might stem from Eisenberger et al. (2002, 2010), who found a supervisor’s organizational embodiment (SOE) to be a moderator of the positive association of LMX with affective organizational commitment. We did not include a moderator, such as SOE, into either of our theoretical models or our empirical investigations, but future research could extend our work and investigate whether SOE moderates the relationship between LMX and OI, which could give practitioners even more working surface. In particular, we expect that a supervisor’s adherence to organizational norms in the form of SOE might affect whether employees identify with him or her or the overall organization and, in turn, show varying degrees of normatively aligned behavior [relatedly, see Ashforth et al. (2007) and Sluss and Ashforth (2007, 2008)].

### Practical Implications

Keeping these limitations in mind, we see two particular practical implications arising out of our research. First and in light of the pivotal role of LMX regarding employees’ OI as well as subsequent CWB and OCB, supervisors should be aware of their important role as proxies for an organization. High-quality interpersonal relationships between supervisors and subordinates are beneficial for the organization as a whole because employees tend to generalize their relationship with their supervisor to the organization and align their behavior toward the organization accordingly. Consequently, managers should invest in developing and maintaining high-quality relationships with their employees. Of course, each supervisor–subordinate dyad can be fairly idiosyncratic in terms of an employee’s understanding of a relationship as of high quality (e.g., Liden et al., 1997; Martin et al., 2016, 2018). In general, the LMX literature strongly focuses on the relationship between leaders and subordinates and thereby rather sparsely discusses specific leader behaviors. Yet, in addition to maintaining high-quality relationships with their employees, supervisors should also *walk the talk* by demonstrating behavioral integrity and providing employees with guidance to foster beneficial and prevent detrimental subordinate behaviors (Dineen et al., 2006). In turn, implementing LMX-focused trainings of leaders appears to be a promising avenue for organizations [relatedly, see Graen et al. (1982)].

Second, employees’ OI deserves attention in its own right. The central role of OI in enhancing beneficial as well as mitigating detrimental behaviors, as judged by the respective organization, in itself suggests that organizations would be well advised to maintain identity-enhancing measures that aim at strengthening employees’ OI [relatedly, see Ashforth and Saks (1996), Chao (2012), and Van Knippenberg (2016)]. Specifically, (1) increasing employees’ feelings of oneness with the organization, (2) providing employees with particular reasons as a basis for their pride in being a member of a specific organization, and, of course, (3) valuing employees as organizational members appear to be promising measures for organizations to fortify this “root construct” (Albert et al., 2000, p. 13) of organizational behavior. In that regard, the Actualizing Social and Personal Identity Resources (ASPIRe) model (Haslam et al., 2003) outlines a workshop-based four-phase intervention and has been empirically demonstrated to be promising (Peters et al., 2013). Specifically, the ASPIRe model offers a practical measure for organizations to develop OI among their employees and, thereby, to eventually foster employees’ beneficial attitudes toward and behaviors at the workplace.

## Conclusion

Violations of organizational norms can have deleterious consequences for organizations as our introductory example of employees stealing painkillers at the US pharmacy company CVS illustrated (Lazarus, 2014). In this research, we showed that high-quality interpersonal relationships with the immediate supervisors can strengthen employees’ OI, thereby leading employees to refrain from CWB and to engage in OCB. In closing, we encourage researchers to corroborate and extend our findings—in addition, we invite managers to be aware of the impact the relationship quality they maintain with their employees can have regarding the extent to which employees identify themselves with the organization as a whole as well as the extent to which they engage in beneficial and detrimental behavior at the workplace.

## Data Availability Statement

The data as well as the corresponding R code regarding this manuscript are available at https://osf.io/p6r3u/ – in case of any question, please contact the corresponding author.

## Ethics Statement

Ethical review and approval was not required for the study on human participants in accordance with the local legislation and institutional requirements. The patients/participants provided their written informed consent to participate in this study.

## Author Contributions

MG contributed substantially to the conception, design, acquisition, analysis, and interpretation of data for the work, drafted the work, revised the work critically for important intellectual content, approved the final version to be published, and agreed to be accountable for all aspects of the work in ensuring that questions related to the accuracy or integrity of any part of the work are appropriately investigated and resolved. MD contributed substantially to the conceptualization, acquisition and interpretation of data, revised the work critically for important intellectual content, approved the final version to be published, and agreed to be accountable for all aspects of the work in ensuring that questions related to the accuracy or integrity of any part of the work are appropriately investigated and resolved. KJ revised the work critically for important intellectual content, approved the final version to be published, and agreed to be accountable for all aspects of the work in ensuring that questions related to the accuracy or integrity of any part of the work are appropriately investigated and resolved. All authors contributed to the article and approved the submitted version.

## Conflict of Interest

The authors declare that the research was conducted in the absence of any commercial or financial relationships that could be construed as a potential conflict of interest.

## Appendix

**TABLE A1:** Zero-order correlations, internal consistencies, and descriptive statistics for study 3.

Variable	*M*_in_	*SD*_in_	*M*_ex_	*SD*_ex_	1	2	3	4	5	6	7
(1) Gender (1 = ♂)	58 ♀, 73 ♂		38 ♀, 45 ♂			–0.17	–0.05	–0.20	–0.13	0.01	0.22*
(2) Age	37.44	09.53	37.36	09.78	–0.11		0.61***	0.06	0.09	0.13	–0.05
(3) Tenure	07.97	05.97	07.95	06.19	0.05	0.56***		0.02	0.17	0.06	0.01
(4) LMX	76.03	18.73	75.01	20.11	–0.12	0.09	0.06	0.92 | 0.93	0.59***	0.48***	–0.13
(5) OI	68.93	20.48	70.25	20.28	–0.03	0.08	0.24**	0.48***	0.95 | 0.96	0.70***	−0.28**
(6) OCB	68.49	18.55	67.80	18.84	0.04	0.04	0.04	0.40***	0.69***	0.88 | 0.88	−0.26*
(7) CWB	10.53	09.51	09.76	09.17	0.12	–0.07	–0.01	−0.20*	−0.40***	−0.31****	0.69 | 0.75

*Recall (including control condition): N = 131; recall (excluding control condition): n = 83. The subscript “in” refers to the full sample including the control condition and the subscript “ex” refers to the subsample excluding the control condition. Correlations for the full sample are presented below and correlations for the subsample are presented above the diagonal. Standardized Cronbach’s alpha coefficients are reported along the diagonal where the first coefficient refers to the full sample. LMX, leader–member exchange; OI, organizational identification; OCB, organizational citizenship behavior; CWB, counterproductive work behavior. *p < 0.05, **p < 0.01,***p < 0.001.*

**TABLE A2:** Zero-order correlations, internal consistencies, and descriptive statistics for study 4.

Variable	*M*_in_	*SD*_in_	*M*_ex_	*SD*_ex_	1	2	3	4	5	6	7
(1) Gender (1 = ♂)	60 ♀, 79 ♂		43 ♀, 48 ♂			–0.05	0.08	–0.02	–0.15	–0.09	0.07
(2) Age	36.76	09.30	37.24	10.03	–0.06		0.61***	–0.09	0.05	0.02	–0.14
(3) Tenure	07.71	08.76	07.54	06.96	0.08	0.43***		–0.05	0.13	0.08	0.01
(4) LMX	60.88	33.71	52.94	37.61	0.02	–0.07	0.00	0.97 | 0.97	0.79***	0.75***	−0.41***
(5) OI	62.64	24.98	58.62	27.23	–0.11	0.07	0.14	0.78***	0.97 | 0.97	0.87***	−0.45***
(6) OCB	62.61	24.66	60.05	27.10	–0.03	0.08	0.08	0.72***	0.84***	0.94 | 0.94	−0.47***
(7) CWB	11.63	12.33	13.11	13.70	0.08	–0.14	0.04	−0.45***	−0.47***	−0.46***	0.83 | 0.84

*Vignette (including control condition): N = 139; vignette (excluding control condition): n = 91. The subscript “in” refers to the full sample including the control condition and the subscript “ex” refers to the subsample excluding the control condition. Correlations for the full sample are presented below and correlations for the subsample are presented above the diagonal. Standardized Cronbach’s alpha coefficients are reported along the diagonal where the first coefficient refers to the full sample. LMX, leader–member exchange; OI, organizational identification; OCB, organizational citizenship behavior; CWB, counterproductive work behavior. *p < 0.05, **p < 0.01, ***p < 0.001.*

## References

[B1] Aguinis H. Bradley K. J. (2014). Best practice recommendations for designing and implementing experimental vignette methodology studies. *Organ. Res. Methods* 17 351–371. 10.1177/1094428114547952

[B2] Aguinis H. Hill N. S. Bailey J. R. (2019). Best practices in data collection and preparation: recommendations for reviewers, editors, and authors. *Organ. Res. Methods* 10.1177/1094428119836485 [Epub ahead of print].

[B3] Al-Atwi A. A. Bakir A. (2014). Relationships between status judgments, identification, and counterproductive behavior. *J. Manag. Psychol.* 29 472–489. 10.1108/JMP-02-2012-0040

[B4] Albert S. Ashforth B. E. Dutton J. E. (2000). Organizational identity and identification: charting new waters and building new bridges. *Acad. Manag. Rev.* 25 13–17. 10.5465/AMR.2000.2791600

[B5] Anand S. Hu J. Liden R. C. Vidyarthi P. R. (2011). “Leader–member exchange: recent research findings and prospects for the future,” in *The SAGE Handbook of Leadership*, eds Bryman A. Collinson D. Grint K. Jackson B. Uhl-Bien M. (London: SAGE Publications), 311–325.

[B6] Anseel F. Lievens F. Schollaert E. Choragwicka B. (2010). Response rates in organizational science, 1995–2008: a meta-analytic review and guidelines for survey researchers. *J. Bus. Psychol.* 25 335–349. 10.1007/s10869-010-9157-6

[B7] Ashforth B. E. Harrison S. H. Corley K. G. (2008). Identification in organizations: an examination of four fundamental questions. *J. Manag.* 34 325–374. 10.1177/0149206308316059

[B8] Ashforth B. E. Mael F. A. (1989). Social identity theory and the organization. *Acad. Manag. Rev.* 14 20–39. 10.5465/AMR.1989.4278999

[B9] Ashforth B. E. Saks A. M. (1996). Socialization tactics: Longitudinal effects on newcomer adjustment. *Acad. Manag. J.* 39 149–178. 10.2307/256634

[B10] Ashforth B. E. Sluss D. M. Saks A. M. (2007). Socialization tactics, proactive behavior, and newcomer learning: Integrating socialization models. *J. Vocat. Behav.* 70 447–462. 10.1016/j.jvb.2007.02.001

[B11] Banks G. C. Batchelor J. H. Seers A. O’Boyle E. H. Pollack J. M. Gower K. (2014). What does team-member exchange bring to the party? A meta-analytic review of team and leader social exchange. *J. Organ. Behav.* 35 273–295. 10.1002/job.1885

[B12] Bauer T. N. Erdogan B. (eds). (2015). *The Oxford Handbook of Leader-Member Exchange.* Oxford: Oxford University Press, 10.1093/oxfordhb/9780199326174.001.0001

[B13] Becker T. E. (2005). Potential problems in the statistical control of variables in organizational research: a qualitative analysis with recommendations. *Organ. Res. Methods* 8 274–289. 10.1177/1094428105278021

[B14] Bennett R. J. Stamper C. L. (2001). “Corporate citizenship and deviancy: a study of discretionary work behavior,” in *International Research in the Business Disciplines: Strategies and Organizations in Transition*, Vol. 3 ed. Galbraith C. S. (Bingley: Emerald), 265–284. 10.1016/S1074-7877(02)03015-5

[B15] Berry C. M. Carpenter N. C. Barratt C. L. (2012). Do other-reports of counterproductive work behavior provide an incremental contribution over self-reports? A meta-analytic comparison. *J. Appl. Psychol.* 97 613–636. 10.1037/a0026739 22201245

[B16] Berry C. M. Ones D. S. Sackett P. R. (2007). Interpersonal deviance, organizational deviance, and their common correlates: a review and meta-analysis. *J. Appl. Psychol.* 92 410–424. 10.1037/0021-9010.92.2.410 17371088

[B17] Blader S. L. Tyler T. R. (2009). Testing and extending the group engagement model: linkages between social identity, procedural justice, economic outcomes, and extrarole behavior. *J. Appl. Psychol.* 94 445–464. 10.1037/a0013935 19271800

[B18] Blanton H. Christie C. (2003). Deviance regulation: a theory of action and identity. *Rev. Gene. Psychol.* 7 115–149. 10.1037/1089-2680.7.2.115

[B19] Blau P. M. (1964). *Exchange and Power in Social Life.* New York, NY: Wiley.

[B20] Bollmann G. Krings F. (2016). Workgroup climates and employees’ counterproductive work behaviours: a social-cognitive perspective. *J. Manag. Stud.* 53 184–209. 10.1111/joms.12167

[B21] Brislin R. W. (1970). Back-translation for cross-cultural research. *J. Cross Cult. Psychol.* 1 185–216. 10.1177/135910457000100301

[B22] Brown T. A. (2015). *Confirmatory Factor Analysis for Applied Research*, 2nd Edn New York, NY: Guilford Press.

[B23] Buhrmester M. D. Kwang T. Gosling S. D. (2011). Amazon’s mechanical turk. *Perspect. Psychol. Sci.* 6 3–5. 10.1177/1745691610393980 26162106

[B24] Buhrmester M. D. Talaifar S. Gosling S. D. (2018). An evaluation of Amazon’s Mechanical Turk, its rapid rise, and its effective use. *Perspect. Psychol. Sci.* 13 149–154. 10.1177/1745691617706516 29928846

[B25] Carmeli A. Atwater L. Levi A. (2011). How leadership enhances employees’ knowledge sharing: the intervening roles of relational and organizational identification. *J. Technol. Trans.* 36 257–274. 10.1007/s10961-010-9154-y

[B26] Carpenter N. C. Rangel B. Jeon G. Cottrell J. M. (2017). Are supervisors and coworkers likely to witness employee counterproductive work behavior? An investigation of observability and self-observer convergence. *Pers. Psychol.* 70 843–889. 10.1111/peps.12210

[B27] Chao G. T. (2012). “Organizational socialization: background, basics, and a blueprint for adjustment at work,” in *The Oxford Handbook of Organizational Psychology*, Vol. 1 ed. Kozlowski S. W. J. (Oxford: Oxford University Press), 579–614. 10.1093/oxfordhb/9780199928309.013.0018

[B28] Cohen J. Cohen P. West S. G. Aiken L. S. (2003). *Applied Multiple Regression/Corrrelation Analysis for The Behavioral Sciences*, 3rd Edn New York, NY: Routledge.

[B29] Cole D. A. Preacher K. J. (2014). Manifest variable path analysis: Potentially serious and misleading consequences due to uncorrected measurement error. *Psychol. Methods* 19 300–315. 10.1037/a0033805 24079927

[B30] Colquitt J. A. Scott B. A. Rodell J. B. Long D. M. Zapata C. P. Conlon D. E. (2013). Justice at the millennium, a decade later: a meta-analytic test of social exchange and affect-based perspectives. *J. Appl. Psychol.* 98 199–236. 10.1037/a0031757 23458336

[B31] Conroy S. A. Henle C. A. Shore L. M. Stelman S. (2017). Where there is light, there is dark: a review of the detrimental outcomes of high organizational identification. *J. Organ. Behav.* 38 184–203. 10.1002/job.2164

[B32] Conway J. M. Lance C. E. (2010). What reviewers should expect from authors regarding common method bias in organizational research. *J. Bus. Psychol.* 25 325–334. 10.1007/s10869-010-9181-6

[B33] Cropanzano R. S. Anthony E. L. Daniels S. R. Hall A. V. (2017). Social exchange theory: a critical review with theoretical remedies. *Acad. Manag. Ann.* 11 479–516. 10.5465/annals.2015.0099

[B34] Cropanzano R. S. Mitchell M. S. (2005). Social exchange theory: an interdisciplinary review. *J. Manag.* 31 874–900. 10.1177/0149206305279602

[B35] Dalal D. K. Hakel M. D. (2016). Experimental comparisons of methods for reducing deliberate distortions to self-report measures of sensitive constructs. *Organ. Res. Methods* 19 475–505. 10.1177/1094428116639131

[B36] Dalal R. S. (2005). A meta-analysis of the relationship between organizational citizenship behavior and counterproductive work behavior. *J. Appl. Psychol.* 90 1241–1255. 10.1037/0021-9010.90.6.1241 16316277

[B37] Dalal R. S. Carpenter N. C. (2018). “The other side of the coin? Similarities and differences between organizational citizenship behavior and counterproductive work behavior,” in *The Oxford Handbook of Organizational Citizenship Behavior*, eds Podsakoff P. M. MacKenzie S. B. Podsakoff N. P. (New York, NY: Oxford University Press), 69–90. 10.1093/oxfordhb/9780190219000.013.4

[B38] De Simone J. A. Harms P. D. (2018). Dirty data: the effects of screening respondents who provide low-quality data in survey research. *J. Bus. Psychol.* 33 559–577. 10.1007/s10869-017-9514-9

[B39] De Simone J. A. Harms P. D. De Simone A. J. (2015). Best practice recommendations for data screening. *J. Organ. Behav.* 36 171–181. 10.1002/job.1962

[B40] Dineen B. R. Lewicki R. J. Tomlinson E. C. (2006). Supervisory guidance and behavioral integrity: relationships with employee citizenship and deviant behavior. *J. Appl. Psychol.* 91 622–635. 10.1037/0021-9010.91.3.622 16737359

[B41] Dukerich J. M. Kramer R. M. Parks J. M. (1998). “The dark side of organizational identification,” in *Identity in Organizations: Building Theory Through Conversations*, eds Whetten D. A. Godfrey P. C. (Thousand Oaks, CA: SAGE Publications), 245–256.

[B42] Dulebohn J. H. Bommer W. H. Liden R. C. Brouer R. L. Ferris G. R. (2012). A meta-analysis of antecedents and consequences of leader-member exchange: integrating the past with an eye toward the future. *J. Manag.* 38 1715–1759. 10.1177/0149206311415280

[B43] Dulebohn J. H. Wu D. Liao C. (2017). Does liking explain variance above and beyond LMX? A meta-analysis. *Hum. Res. Manag. Rev.* 27 149–166. 10.1016/j.hrmr.2016.09.008

[B44] Eisenberger R. Karagonlar G. Stinglhamber F. Neves P. Becker T. E. Gonzalez-Morales M. G. (2010). Leader–member exchange and affective organizational commitment: the contribution of supervisor’s organizational embodiment. *J. Appl. Psychol.* 95 1085–1103. 10.1037/a0020858 20718516

[B45] Eisenberger R. Rockstuhl T. Shoss M. K. Wen X. Dulebohn J. (2019). Is the employee–organization relationship dying or thriving? A temporal meta-analysis. *J. Appl. Psychol.* 104 1036–1057. 10.1037/apl0000390 30730164

[B46] Eisenberger R. Stinglhamber F. (2011). *Perceived Organizational Support: Fostering Enthusiastic and Productive Employees.* Washington, DC: American Psychological Association, 10.1037/12318-000

[B47] Eisenberger R. Stinglhamber F. Vandenberghe C. Sucharski I. L. Rhoades L. (2002). Perceived supervisor support: Contributions to perceived organizational support and employee retention. *J. Appl. Psychol.* 87 565–573. 10.1037/0021-9010.87.3.565 12090614

[B48] Epitropaki O. Martin R. Thomas G. (2018). “Relational leadership,” in *The Nature of Leadership*, 3rd Edn, eds Antonakis J. Day D. V. (Thousand Oaks, CA: SAGE Publications), 10–137.

[B49] Evans W. R. Davis W. (2014). Corporate citizenship and the employee: an organizational identification perspective. *Hum. Perform.* 27 129–146. 10.1080/08959285.2014.882926

[B50] Gerstner C. R. Day D. V. (1997). Meta-analytic review of leader-member exchange theory: correlates and construct issues. *J. Appl. Psychol.* 82 827–844. 10.1037/0021-9010.82.6.827

[B51] Gleibs I. H. (2017). Are all “research fields” equal? Rethinking practice for the use of data from crowdsourcing market places. *Behav. Res. Methods* 49 1333–1342. 10.3758/s13428-016-0789-y 27515317 PMC5541108

[B52] Gooty J. Yammarino F. J. (2016). The leader–member exchange relationship: a multisource, cross-level investigation. *J. Manag.* 42 915–935. 10.1177/0149206313503009

[B53] Götz M. Bollmann G. O’Boyle E. H. (2019). Contextual undertow of workplace deviance by and within units: a systematic review. *Small Group Res.* 50 39–80. 10.1177/1046496418790044

[B54] Gouldner A. W. (1960). The norm of reciprocity: a preliminary statement. *Am. Sociol. Rev.* 25:161 10.2307/2092623

[B55] Graen G. B. Novak M. A. Sommerkamp P. (1982). The effects of leader—member exchange and job design on productivity and satisfaction: testing a dual attachment model. *Organ. Behav. Hum. Perform.* 30 109–131. 10.1016/0030-5073(82)90236-7

[B56] Graen G. B. Uhl-Bien M. (1995). Relationship-based approach to leadership: development of leader-member exchange (LMX) theory of leadership over 25 years: applying a multi-level multi-domain perspective. *Leadership Q.* 6 219–247. 10.1016/1048-9843(95)90036-5

[B57] Greco L. M. O’Boyle E. H. Walter S. L. (2015). Absence of malice: a meta-analysis of nonresponse bias in counterproductive work behavior research. *J. Appl. Psychol.* 100 75–97. 10.1037/a0037495 25089858

[B58] Greco L. M. Whitson J. A. O’Boyle E. H. Wang C. S. Kim J. (2019). An eye for an eye? A meta-analysis of negative reciprocity in organizations. *J. Appl. Psychol.* 104 1117–1143. 10.1037/apl0000396 30762379

[B59] Haslam S. A. (2004). *Psychology in Organizations: The Social Identity Approach*, 2nd Edn London: SAGE Publications.

[B60] Haslam S. A. Eggins R. A. Reynolds K. J. (2003). The ASPIRe model: actualizing social and personal identity resources to enhance organizational outcomes. *J. Occupat. Organ. Psychol.* 76 83–113. 10.1348/096317903321208907 30467716

[B61] Haslam S. A. Ellemers N. (2006). “Social identity in industrial and organizational psychology: concepts, controversies and contributions,” in *International Review of Industrial Organisational Psychology.* Vol. 20 eds Hodgkinson G. P. Ford J. K. (Chichester: John Wiley & Sons), 39–118. 10.1002/0470029307.ch2

[B62] Haslam S. A. Turner J. C. Oakes P. J. McGarty C. A. Reynolds K. J. (1997). The group as a basis for emergent stereotype consensus. *Eur. Rev. Soc. Psychol.* 8 203–239. 10.1080/14792779643000128

[B63] Henze N. Zirkler B. (1990). A class of invariant consistent tests for multivariate normality. *Commun. Statist. Theory Methods* 19 3595–3617. 10.1080/03610929008830400

[B64] Hu L. Bentler P. M. (1999). Cutoff criteria for fit indexes in covariance structure analysis: conventional criteria versus new alternatives. *Struct. Equat. Model.* 6 1–55. 10.1080/10705519909540118

[B65] Huang J. L. Bowling N. A. Liu M. Li Y. (2015). Detecting insufficient effort responding with an infrequency scale: evaluating validity and participant reactions. *J. Bus. Psychol.* 30 299–311. 10.1007/s10869-014-9357-6

[B66] Huang J. L. Curran P. G. Keeney J. Poposki E. M. DeShon R. P. (2012). Detecting and deterring insufficient effort responding to surveys. *J. Bus. Psychol.* 27 99–114. 10.1007/s10869-011-9231-8

[B67] Hunt S. T. (1996). Generic work behavior: an investigation into the dimensions of entry−level, hourly job performance. *Pers. Psychol.* 49 51–83. 10.1111/j.1744-6570.1996.tb01791.x

[B68] Katrinli A. Atabay G. Gunay G. Guneri B. (2008). Leader-member exchange, organizational identification and the mediating role of job involvement for nurses. *J. Adv. Nurs.* 64 354–362. 10.1111/j.1365-2648.2008.04809.x 18990114

[B69] Kelman H. C. (1958). Compliance, identification, and internalization three processes of attitude change. *J. Conf. Resolut.* 2 51–60. 10.1177/002200275800200106

[B70] Klein H. J. Molloy J. C. Brinsfield C. T. (2012). Reconceptualizing workplace commitment to redress a stretched construct: revisiting assumptions and removing confounds. *Acad. Manag. Rev.* 37 130–151. 10.5465/arma.2010.0018

[B71] Klein K. J. Palmer S. L. Conn A. B. (2000). “Interorganizational relationships: a multilevel perspective,” in *Multilevel Theory, Research, and Methods in Organizations: Foundations, Extensions, and New Directions*, eds Klein K. J. Kozlowski S. W. J. (San Francisco, CA: Jossey-Bass), 267–307.

[B72] Kline R. B. (2015). The mediation myth. *Basic Appl. Soc. Psychol.* 37 202–213. 10.1080/01973533.2015.1049349

[B73] Kline R. B. (2016). *Principles and Practice of Structural Equation Modeling*, 4th Edn New York, NY: Guilford Press.

[B74] Korkmaz S. Goksuluk D. Zararsiz G. (2014). MVN: An R package for assessing multivariate normality. *R J.* 6 151–162. 10.32614/RJ-2014-031

[B75] Kuhlmann T. Dantlgraber M. Reips U.-D. (2017). Investigating measurement equivalence of visual analogue scales and Likert-type scales in Internet-based personality questionnaires. *Behav. Res. Methods* 49 2173–2181. 10.3758/s13428-017-0868-8 28130728

[B76] Landis R. S. Beal D. J. Tesluk P. E. (2000). A comparison of approaches to forming composite measures in structural equation models. *Organ. Res. Methods* 3 186–207. 10.1177/109442810032003

[B77] Landis R. S. Edwards B. D. Cortina J. M. (2009). “On the practice of allowing correlated residuals among indicators in structural equation models,” in *Statistical and Methodological Myths and Urban Legends: Doctrine, Verity and Fable in the Organizational and Social Sciences*, eds Lance C. E. Vandenberg R. J. (New York, NY: Routledge), 193–215. 10.4324/9780203867266

[B78] Lavelle J. J. Rupp D. E. Brockner J. (2007). Taking a multifoci approach to the study of justice, social exchange, and citizenship behavior: the target similarity model. *J. Manag.* 33 841–866. 10.1177/0149206307307635

[B79] Lazarus D. (2014). *U.S. Should Reclassify Prescription Painkiller to cut Rampant Theft.* Los Angeles, CA: Los Angeles Times.

[B80] Lee E.-S. Park T.-Y. Koo B. (2015). Identifying organizational identification as a basis for attitudes and behaviors: A meta-analytic review. *Psychol. Bullet.* 141 1049–1080. 10.1037/bul0000012 25984729

[B81] Leiner D. J. (2019). *SoSci Survey [Computer Software]*. Available online at: https://www.soscisurvey.de/

[B82] Liao H. Joshi A. Chuang A. (2004). Sticking out like a sore thumb: employee dissimilarity and deviance at work. *Pers. Psychol.* 57 969–1000. 10.1111/j.1744-6570.2004.00012.x

[B83] Liden R. C. Maslyn J. M. (1998). Multidimensionality of leader-member exchange: an empirical assessment through scale development. *J. Manag.* 24 43–72. 10.1177/014920639802400105

[B84] Liden R. C. Sparrowe R. T. Wayne S. J. (1997). “Leader-member exchange theory: the past and potential for the future,” in *Research in Personnel and Human Resources Management*, Vol. 15 ed. Ferris G. R. (Bingley: JAI Press), 47–119.

[B85] Little T. D. Cunningham W. A. Shahar G. Widaman K. F. (2002). To parcel or not to parcel: exploring the question, weighing the merits. *Struct. Equat. Model.* 9 233–255. 10.1207/S15328007SEM0902

[B86] Liu Y. Mo S. Song Y. Wang M. (2016). Longitudinal analysis in occupational health psychology: a review and tutorial of three longitudinal modeling techniques. *Appl. Psychol.* 65 379–411. 10.1111/apps.12055

[B87] Loi R. Chan K. W. Lam L. W. (2014). Leader-member exchange, organizational identification, and job satisfaction: a social identity perspective. *J. Occup. Organ. Psychol.* 87 42–61. 10.1111/joop.12028

[B88] Mael F. A. Ashforth B. E. (1992). Alumni and their alma mater: a partial test of the reformulated model of organizational identification. *J. Organ. Behav.* 13 103–123. 10.1002/job.4030130202

[B89] Marcus B. Taylor O. A. Hastings S. E. Sturm A. Weigelt O. (2016). The structure of counterproductive work behavior: a review, a structural meta-analysis, and a primary study. *J. Manag.* 42 203–233. 10.1177/0149206313503019

[B90] Martin R. Epitropaki O. Thomas G. Topakas A. (2010). “A review of leader–member exchange research: future prospects and sirections,” in *International review of industrial organisational psychology*, Vol. 25 eds Gerard P. H. Ford J. K. (Chichester: John Wiley & Sons), 35–88. 10.1002/9780470661628.ch2

[B91] Martin R. Guillaume Y. R. F. Thomas G. Lee A. Epitropaki O. (2016). Leader-member exchange (LMX) and performance: a meta-analytic review. *Pers. Psychol.* 69 67–121. 10.1111/peps.12100

[B92] Martin R. Thomas G. Legood A. Dello Russo S. (2018). Leader-member exchange (LMX) differentiation and work outcomes: conceptual clarification and critical review. *J. Organ. Behav.* 39 151–168. 10.1002/job.2202

[B93] Maxwell J. C. (2011). *The 360 Degree Leader: Developing Your Influence From Anywhere in the Organization.* Nashville, TN: Thomas Nelson.

[B94] Mercado B. K. Dilchert S. Giordano C. Ones D. S. (2018). “Counterproductive work behaviors,” in *The SAGE Handbook of Industrial, Work and Organizational Psychology: Personnel Psychology and Employee Performance*, 2nd Edn, eds Ones D. S. Anderson N. Viswesvaran C. Sinangil H. K. (London: SAGE Publications), 109–211.

[B95] Norman S. M. Avey J. B. Nimnicht J. L. Graber Pigeon N. (2010). The interactive effects of psychological capital and organizational identity on employee organizational citizenship and deviance behaviors. *J. Leader. Organ. Stud.* 17 380–391. 10.1177/1548051809353764

[B96] O’Boyle E. H. Forsyth D. R. O’Boyle A. S. (2011). Bad apples or bad barrels: an examination of group- and organizational-level effects in the study of counterproductive work behavior. *Group Organ. Manag.* 36 39–69. 10.1177/1059601110390998

[B97] O’Laughlin K. D. Martin M. J. Ferrer E. (2018). Cross-sectional analysis of longitudinal mediation processes. *Multiv. Behav. Res.* 53 375–402. 10.1080/00273171.2018.1454822 29624079

[B98] Organ D. W. (1988). *Organizational Citizenship Behavior: The Good Soldier Syndrome.* Lexington, MA: Lexington Books.

[B99] Organ D. W. Podsakoff P. M. MacKenzie S. B. (2006). *Organizational Citizenship Behavior: Its Nature, Antecedents, and Consequences.* Thousand Oaks, CA: SAGE Publications.

[B100] Ostroff C. (2019). “Contextualizing context in organizational research,” in *The Handbook of Multilevel Theory, Measurement, and Analysis*, eds Humphrey S. E. LeBreton J. M. (Washington, DC: American Psychological Association), 39–65. 10.1037/0000115-003

[B101] Palmer D. A. (2012). *Normal Organizational Wrongdoing: A Critical Analysis of Theories of Misconduct in and by Organizations.* Oxford: Oxford University Press.

[B102] Pan J. Ip E. H. Dubé L. (2017). An alternative to post hoc model modification in confirmatory factor analysis: the Bayesian lasso. *Psychol. Methods* 22 687–704. 10.1037/met0000112 29265848 PMC5745070

[B103] Paulhus D. L. (1984). Two-component models of socially desirable responding. *J. Pers. Soc. Psychol.* 46 598–609. 10.1037/0022-3514.46.3.598

[B104] Peer E. Vosgerau J. Acquisti A. (2014). Reputation as a sufficient condition for data quality on Amazon Mechanical Turk. *Behav. Res. Methods* 46 1023–1031. 10.3758/s13428-013-0434-y 24356996

[B105] Peters K. Haslam S. A. Ryan M. K. Fonseca M. (2013). Working with subgroup identities to build organizational identification and support for organizational strategy: a test of the ASPIRe model. *Group Organ. Manag.* 38 128–144. 10.1177/1059601112472368

[B106] Pirlott A. G. MacKinnon D. P. (2016). Design approaches to experimental mediation. *J. Exp. Soc. Psychol.* 66 29–38. 10.1016/j.jesp.2015.09.012 27570259 PMC4999253

[B107] Ployhart R. E. MacKenzie W. I. Jr. (2015). “Two waves of measurement do not a longitudinal study make,” in *More Statistical and Methodological Myths and Urban Legends*, eds Lance C. E. Vandenberg R. J. (New York, NY: Routledge), 85–99.

[B108] Podsakoff P. M. Mackenzie S. B. Podsakoff N. P. (eds). (2018). *The Oxford Handbook of Organizational Citizenship Behavior.* New York, NY: Oxford University Press, 10.1093/oxfordhb/9780190219000.001.0001

[B109] Podsakoff P. M. MacKenzie S. B. Podsakoff N. P. (2012). Sources of method bias in social science research and recommendations on how to control it. *Ann. Rev. Psychol.* 63 539–569. 10.1146/annurev-psych-120710-100452 21838546

[B110] Podsakoff P. M. Podsakoff N. P. (2019). Experimental designs in management and leadership research: strengths, limitations, and recommendations for improving publishability. *Leader. Q.* 30 11–33. 10.1016/j.leaqua.2018.11.002

[B111] Porter C. O. L. H. Outlaw R. Gale J. P. Cho T. S. (2019). The use of online panel data in management research: a review and recommendations. *J. Manag.* 45 319–344. 10.1177/0149206318811569

[B112] R Development Core Team (2020). *R: A Language and Environment for Statistical Computing.* Vienna: R Development Core Team.

[B113] Rausch M. Zehetleitner M. (2014). A comparison between a visual analogue scale and a four point scale as measures of conscious experience of motion. *Conscious. Cogn.* 28 126–140. 10.1016/j.concog.2014.06.012 25058629

[B114] Reips U.-D. Funke F. (2008). Interval-level measurement with visual analogue scales in Internet-based research: VAS Generator. *Behav. Res. Methods* 40 699–704. 10.3758/BRM.40.3.699 18697664

[B115] Rhoades L. Eisenberger R. (2002). Perceived organizational support: a review of the literature. *J. Appl. Psychol.* 87 698–714. 10.1037//0021-9010.87.4.698 12184574

[B116] Riketta M. (2005). Organizational identification: a meta-analysis. *J. Vocat. Behav.* 66 358–384. 10.1016/j.jvb.2004.05.005

[B117] Riketta M. Van Dick R. (2005). Foci of attachment in organizations: a meta-analytic comparison of the strength and correlates of workgroup versus organizational identification and commitment. *J. Vocat. Behav.* 67 490–510. 10.1016/j.jvb.2004.06.001

[B118] Robinson S. L. Bennett R. J. (1995). A typology of deviant workplace behaviors: a multidimensional scaling study. *Acad. Manag. J.* 38 555–572. 10.2307/256693

[B119] Robinson S. L. Kraatz M. S. (1998). “Constructing the reality of normative behavior: the use of neutralization strategies by organizational deviants,” in *Dysfunctional Behavior in Organizations, Part A: Violent and Deviant Behavior*, eds Griffin R. W. O’Leary-Kelly A. Collins J. M. (Stamford, CT: JAI Press), 203–220.

[B120] Rosseel Y. (2012). Lavaan: an R package for structural equation modeling. *J. Statist. Softw.* 48 1–36. 10.18637/jss.v048.i02

[B121] Rotundo M. Sackett P. R. (2002). The relative importance of task, citizenship, and counterproductive performance to global ratings of job performance: a policy-capturing approach. *J. Appl. Psychol.* 87 66–80. 10.1037//0021-9010.87.1.66 11916217

[B122] Rousseau D. M. (1995). *Psychological Contracts in Organizations: Understanding Written and Unwritten Agreements.* Thousand Oaks, CA: SAGE Publications.

[B123] Saris W. E. Gallhofer I. N. (2014). *Design, Evaluation, and Analysis of Questionnaires for Survey Research*, 2nd Edn Hoboken, NJ: John Wiley & Sons.

[B124] Scandura T. A. Graen G. B. (1984). Moderating effects of initial leader–member exchange status on the effects of a leadership intervention. *J. Appl. Psychol.* 69 428–436. 10.1037/0021-9010.69.3.428

[B125] Schriesheim C. A. Castro S. L. Cogliser C. C. (1999). Leader-member exchange (LMX) research: a comprehensive review of theory, measurement, and data-analytic practices. *Leader. Q.* 10 63–113. 10.1016/S1048-9843(99)80009-5

[B126] Schyns B. (2002). Überprüfung einer deutschsprachigen Skala zum Leader-Member-Exchange-Ansatz [Evaluation of a German scale for the assessment of leader-member exchange]. *Zeitschr. Differ. Diagnos. Psychol.* 23 235–245. 10.1024//0170-1789.23.2.235

[B127] Seo J. J. Nahrgang J. D. Carter M. Z. Hom P. W. (2018). Not all differentiation is the same: Examining the moderating effects of leader-member exchange (LMX) configurations. *J. Appl. Psychol.* 103 478–495. 10.1037/apl0000262 29239643

[B128] Shadish W. R. Cook T. D. Campbell D. T. (2002). *Experimental and Quasi-Experimental Designs for Generalized Causal Inference.* Boston, MA: Houghton Mifflin.

[B129] Sluss D. M. Ashforth B. E. (2007). Relational identity and identification: defining ourselves through work relationships. *Acad. Manag. Rev.* 32 9–32. 10.5465/AMR.2007.23463672

[B130] Sluss D. M. Ashforth B. E. (2008). How relational and organizational identification converge: processes and conditions. *Organ. Sci.* 19 807–823. 10.1287/orsc.1070.0349 19642375

[B131] Spector P. E. (2019). Do not cross me: optimizing the use of cross-sectional designs. *J. Bus. Psychol.* 34 125–137. 10.1007/s10869-018-09613-8

[B132] Spector P. E. Bauer J. A. Fox S. (2010). Measurement artifacts in the assessment of counterproductive work behavior and organizational citizenship behavior: do we know what we think we know? *J. Appl. Psychol.* 95 781–790. 10.1037/a0019477 20604597

[B133] Spector P. E. Che X. X. (2014). Re-examining citizenship: How the control of measurement artifacts affects observed relationships of organizational citizenship behavior and organizational variables. *Hum. Perform.* 27 165–182. 10.1080/08959285.2014.882928

[B134] Spector P. E. Fox S. (2010). Counterproductive work behavior and organisational citizenship behavior: are they opposite forms of active behavior? *Appl. Psychol.* 59 21–39. 10.1111/j.1464-0597.2009.00414.x 20604597

[B135] Spencer S. J. Zanna M. P. Fong G. T. (2005). Establishing a causal chain: why experiments are often more effective than mediational analyses in examining psychological processes. *J. Pers. Soc. Psychol.* 89 845–851. 10.1037/0022-3514.89.6.845 16393019

[B136] Spitzmüller M. Ilies R. Choi D. (2018). “Organizational citizenship behaviors–A new look at an old phenomenon at different levels,” in *The SAGE Handbook of Industrial, Work and Organizational Psychology: Personnel Psychology and Employee Performance*, 2nd Edn, eds Ones D. S. Anderson N. Viswesvaran C. Sinangil H. K. (London: SAGE Publications), 89–108.

[B137] Stone-Romero E. F. Rosopa P. J. (2008). The relative validity of inferences about mediation as a function of research design characteristics. *Organ. Res. Methods* 11 326–352. 10.1177/1094428107300342

[B138] Tajfel H. (1981). *Human Groups and Social Categories: Studies in Social Psychology.* Cambridge: Cambridge University Press.

[B139] Tajfel H. Turner J. C. (1986). “The social identity theory of intergroup behavior,” in *Psychology of Intergroup Relations*, 2nd Edn, eds Worchel S. Austin L. W. (Chigago, IL: Nelson-Hall), 8–24.

[B140] Tofighi D. MacKinnon D. P. (2011). RMediation: an R package for mediation analysis confidence intervals. *Behav. Res. Methods* 43 692–700. 10.3758/s13428-011-0076-x 21487904 PMC3233842

[B141] Tourangeau R. Yan T. (2007). Sensitive questions in surveys. *Psychol. Bull.* 133 859–883. 10.1037/0033-2909.133.5.859 17723033

[B142] Turner J. C. Hogg M. A. Oakes P. J. Reicher S. D. Wetherell M. S. (1987). *Rediscovering the Social Group: A self-Categorization Theory.* Oxford: Blackwell Publishing.

[B143] Turner S. F. Cardinal L. B. Burton R. M. (2017). Research design for mixed methods: a triangulation-based framework and roadmap. *Organ. Res. Methods* 20 243–267. 10.1177/1094428115610808

[B144] Tyler T. R. Blader S. L. (2003). The group engagement model: procedural justice, social identity, and cooperative behavior. *Pers. Soc. Psychol. Rev.* 7 349–361. 10.1207/S15327957PSPR0704_07 14633471

[B145] Umphress E. E. Bingham J. B. (2011). When employees do bad things for good reasons: examining unethical pro-organizational behaviors. *Organ. Sci.* 22 621–640. 10.1287/orsc.1100.0559 19642375

[B146] Umphress E. E. Bingham J. B. Mitchell M. S. (2010). Unethical behavior in the name of the company: The moderating effect of organizational identification and positive reciprocity beliefs on unethical pro-organizational behavior. *J. Appl. Psychol.* 95 769–780. 10.1037/a0019214 20604596

[B147] Vadera A. K. Pratt M. G. (2013). Love, hate, ambivalence, or indifference? A conceptual examination of workplace crimes and organizational identification. *Organ. Sci.* 24 172–188. 10.1287/orsc.1110.0714 19642375

[B148] Van Knippenberg D. (2016). “Making sense of who we are: leadership and organizational identity,” in *The Oxford Handbook of Organizational Identity*, eds Pratt M. G. Schultz M. Ashforth B. E. Ravasi D. (Oxford: Oxford University Press), 335–349.

[B149] Van Knippenberg D. Sleebos E. (2006). Organizational identification versus organizational commitment: self-definition, social exchange, and job attitudes. *J. Organ. Behav.* 27 571–584. 10.1002/job.359

[B150] Viswesvaran C. Ones D. S. (2000). Perspectives on models of job performance. *Int. J. Select. Assess.* 8 216–226. 10.1111/1468-2389.00151

[B151] Warren D. E. (2003). Constructive and destructive deviance in organizations. *Acad. Manag. Rev.* 28 622–632. 10.5465/AMR.2003.10899440

[B152] Wheeler A. R. Shanine K. K. Leon M. R. Whitman M. V. (2014). Student-recruited samples in organizational research: a review, analysis, and guidelines for future research. *J. Occup. Organ. Psychol.* 87 1–26. 10.1111/joop.12042

[B153] Williams L. J. O’Boyle E. H. (2008). Measurement models for linking latent variables and indicators: a review of human resource management research using parcels. *Hum. Resour. Manag. Rev.* 18 233–242. 10.1016/j.hrmr.2008.07.002

[B154] Yam K. C. Klotz A. C. He W. Reynolds S. J. (2017). From good soldiers to psychologically entitled: examining when and why citizenship behavior leads to deviance. *Acad. Manag. J.* 60 373–396. 10.5465/amj.2014.0234

[B155] Yuan K.-H. Bentler P. M. (1998). Structural equation modeling with robust covariances. *Soc. Methodol.* 28 363–396. 10.1111/0081-1750.00052

[B156] Zhang Y. Chen C. C. (2013). Developmental leadership and organizational citizenship behavior: Mediating effects of self-determination, supervisor identification, and organizational identification. *Leaders. Q.* 24 534–543. 10.1016/j.leaqua.2013.03.007

[B157] Zhao H. Liu W. Li J. Yu X. (2019). Leader–member exchange, organizational identification, and knowledge hiding: the moderating role of relative leader–member exchange. *J. Organ. Behav.* 40 834–848. 10.1002/job.2359

[B158] Zhou H. Fishbach A. (2016). The pitfall of experimenting on the web: how unattended selective attrition leads to surprising (yet false) research conclusions. *J. Pers. Soc. Psychol.* 111 493–504. 10.1037/pspa0000056 27295328

